# Metastatic Phosphatase PRL-3 Induces Ovarian Cancer Stem Cell Sub-population through Phosphatase-Independent Deacetylation Modulations

**DOI:** 10.1016/j.isci.2019.100766

**Published:** 2019-12-12

**Authors:** Mingming Zhang, Yanli Wei, Yanbin Liu, Wen Guan, Xiaomei Zhang, Jianqiu Kong, Hui Li, Shulan Yang, Haihe Wang

**Affiliations:** 1 Centre for Translational Medicine, the First Affiliated Hospital, Sun Yat-sen University, Guangzhou 510080, China; 2 Department of Biochemistry, Zhongshan School of Medicine, Sun Yat-sen University, Guangzhou 510080, China; 3 Institute of Immunology and Molecular Medicine, Jining Medical University, Jining, Shandong 272067, China; 4 Guangdong Engineering & Technology Research Center for Disease-Model Animals, Sun Yat-sen University, Guangzhou 510006, China; 5 Center for Stem Cell Biology and Tissue Engineering, Key Laboratory of Ministry of Education, Sun Yat-sen University, Guangzhou 510080, China

**Keywords:** Biological Sciences, Molecular Biology, Stem Cells Research, Cancer

## Abstract

Cancer stem cells (CSCs) are responsible for tumor initiation, chemoresistance, metastasis, and relapse, but the underlying molecular origin of CSCs remains elusive. Here we identified that metastatic phosphatase of regenerating liver 3 (PRL-3) transcriptionally upregulates SOX2 in the expansion of CSC sub-population from normal cancer cells. Mechanistically, SOX2 upregulation is attributed to the binding of the acetylated myocyte enhancer factor 2A (MEF2A) to SOX2 promoter in tumor cells. In parallel, PRL-3 competitively binds to Class IIa histone deacetylase 4 (HDAC4) to facilitate HDAC4 translocation, leading to the disassociation of HDAC4 from MEF2A and histones. The released MEF2A and histones thus remain acetylated and render the subsequent accessibility of the acetylated MEF2A to SOX2 promoter region. Clinical relevance among PRL-3, SOX2, and HDAC4 is validated in ovary cancer samples. Therefore, this PRL-3-HDAC4-MEF2A/histones-SOX2 signaling axis would be a potential therapeutic target in inhibiting ovarian cancer metastasis and relapse.

## Highlights

• PRL-3 promotes the expansion of CSC-like cells via transcriptional SOX2 upregulation
• Binding of MEF2A to SOX2 promoter bridges the PRL-3-induced SOX2 upregulation
• PRL-3 competitively binds HDAC4 to cause the disassociation of HDAC4 from MEF2A
• Acetylated histones render the accessibility of SOX2 promoter region to MEF2A

## Graphical Abstract

**Figure:**
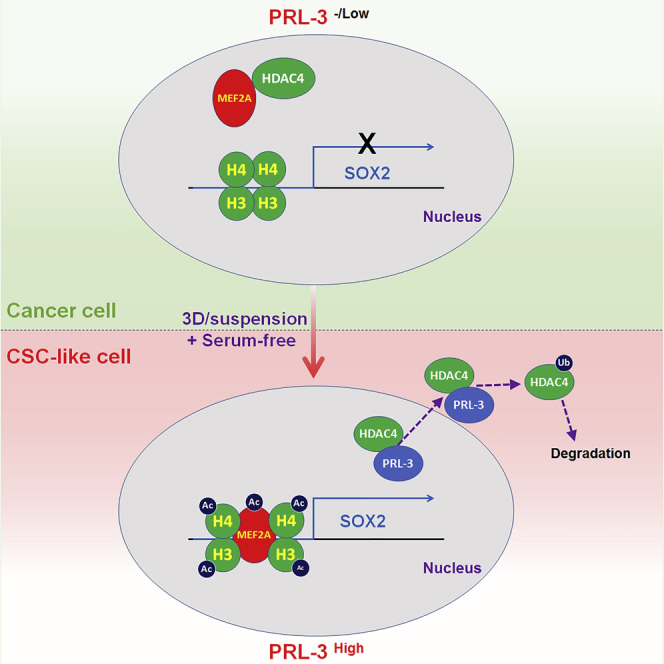

## Introduction

Acquisition of extra stem cell-like features by cancer cells greatly limits the clinical utility of most anticancer drugs. Relapse is also driven by this small sub-population of cells, although most of the tumor cells are rapidly killed upon drug exposure. Recent evidence indicates that the emergence of relapse is unlikely due to all the mutation events in tumor cells (Menon et al., 2015, Sharma et al., 2010), but in part to the occurrence and enrichment of this small sub-population of cancer (stem) cells that is intrinsically heterogeneous and refractory to anti-cancer drugs (Roesch et al., 2013, Trumpp and Wiestler, 2008). This non-mutual scenario proposes that there are non-mutational transition mechanisms under cancer cells to obtain the native or acquired drug tolerance (Ravindran Menon et al., 2015, Sharma et al., 2010, Su et al., 2017). However, the underlying mechanism of this heterogeneous cancer cell state's transition/plasticity remains elusive.

Epithelial ovarian cancer is the most deadly gynecologic malignancy; patients undergoing routine surgery and chemotherapy often suffer from recurrence of disease as the treatment becomes ineffective and tumor migrates to metastatic sites (Vetter and Hays, 2018). Phosphatase of regenerating liver 3 (PRL-3) is found upregulated in metastasis sites of various cancers, and a higher PRL-3 level is related to poor prognosis in several cancers including colon, ovarian, and breast cancer and leukemia (Al-Aidaroos and Zeng, 2010, Peng et al., 2004). Beside its phosphatase function, PRL-3 is also involved in several crucial pathways for carcinogenesis as a multifunctional molecule. Studies already manifest that PRL-3 modifies cell growth, migration, and invasion through Rho, vascular endothelial growth factor, and KCNN4 in different cell lines (Fiordalisi et al., 2006, Zimmerman et al., 2014, Lai et al., 2011). More importantly, PRL-3 causes epithelial-mesenchymal transition (EMT), a key step that leads to cell invasion in carcinogenesis, by inhibiting PTEN expression and activating PI3K-AKT signaling (Wang et al., 2007). Given that EMT and PTEN downregulation are important in breast cancer stem cell (CSC) formation and drug resistance (Sun et al., 2016), we suspect that PRL-3 may promote the transition of cancer cells to CSCs. In line with this hypothesis, it is documented that PRL-3 renders chemoresistance in acute myeloid leukemia (AML) (Zhou et al., 2011). However, whether PRL-3 really plays a driving role in CSC formation remains unknown.

In this study, we disclose that PRL-3 plays a switchable role in tumor cell plasticity of ovarian cancer cells. In this process, PRL-3 enhances SOX2 upregulation via epigenetic modulations of both a transcription factor, MEF2A, and histones H3 and H4 for the efficient MEF2A binding to SOX2 promoter.

## Results

### PRL-3 Enhances the Cell State Transition of Normal Ovarian Cancer Cells to CSC State

**Figure 1:**
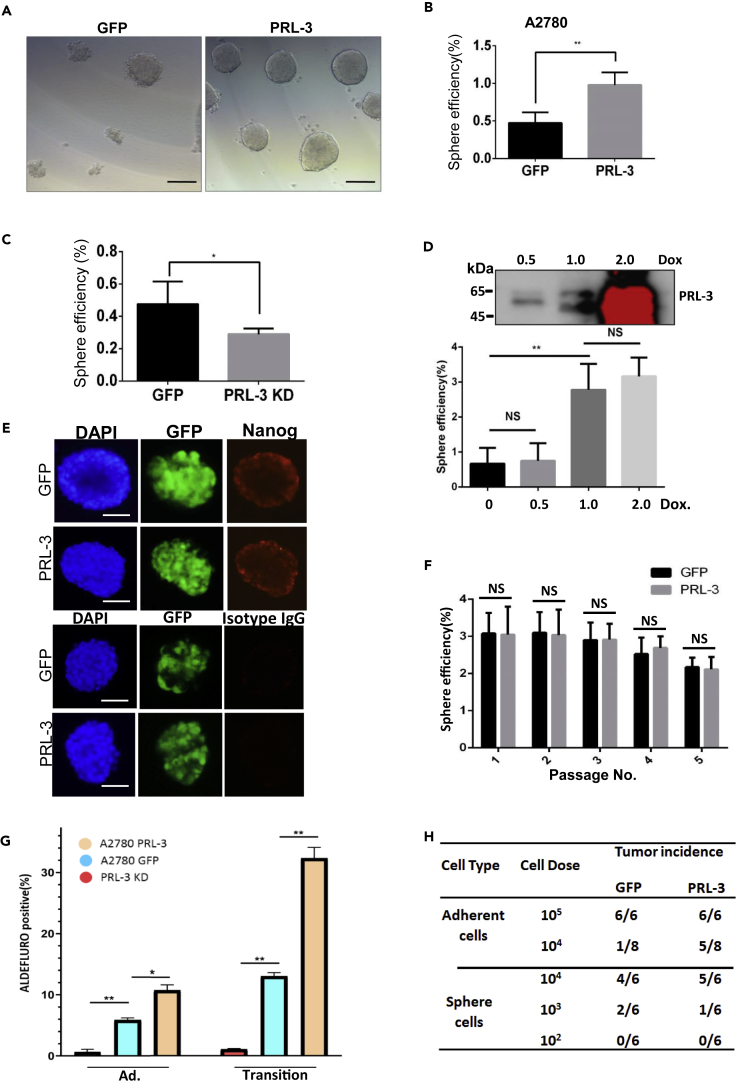
PRL-3 Enhances the Cell State Transition of Normal Ovarian Cancer Cells to CSC (A) Tumor cell spheres formed from both GFP parental and PRL-3-overexpressing cells; 5,000 cells were seeded in six-well plate pre-treated with poly(2-hydroxyethyl methacrylate) coating to prevent cell attachment. Representative images were taken after 5 days induction. (B) Sphere formation efficiency of cells in (A). Tumor spheres were counted and sphere efficiency was calculated as in Transparent Methods section. The assay was performed in triplicate; data are represented as mean ± SEM, ∗∗p < 0.01, unpaired *t* test. (C) Tumor cell spheres formed by A2780 and A2780 PRL-3 KD cells. The induction condition and sphere efficiency were similarly conducted as (A) and (B), respectively. ∗p < 0.05, unpaired *t* test. (D) Cell sphere formation in PRL-3-inducible system of CHO cells. PRL-3 expression was induced by doxycycline (Dox) at the indicated concentrations (upper panel). The induction condition and sphere efficiency were similarly conducted as (A) and (B), respectively. ∗∗p < 0.01, unpaired *t* test. (E) Immunofluorescence staining of tumor spheres from A2780 GFP and A2780 PRL-3 cells to stain Nanog expression. Spheres were fixed with 4% paraformaldehyde and analyzed by Olympus BX63. The isotype mouse kappa light chain antibody (IgG) was used as a negative control. Scale bar, 100 μm. (F) Sphere passage was conducted with the re-dispersed cells from spheres formed. Individual cell from each type of spheres was seeded to check the sphere formation efficiency. Every 5 days were counted as a “passage.” The first batch of spheres was designed as passage “0,” the second as passage “1,” and so on. (G) ALDEFLUOR Assay of A2780 GFP or A2780 PRL-3 cells under adherent culture condition (Ad.) or in the middle of suspension sphere formation stage (Transition). Amount of fluorescent product is proportional to the ALDH activity in the cells and is measured using a CytoFLEX flow cytometer. Data are represented as mean ± SEM, ∗p < 0.05, ∗∗p < 0.01, unpaired *t* test. (H) Xenograft of tumor formation by A2780 GFP and A2780 PRL-3 cells. The indicated number of cells (cell dose) was subcutaneously implanted into flanks of NOD/SCID mice. Tumor incidence (number of mice with formed tumor/number of mice inoculated) was indicated as an index for tumor formation ability.

To directly investigate whether PRL-3 functions in the formation of “stem-like” cancer cells, we first forced expressed GFP-PRL-3 fusion protein in human ovarian cancer cell lines A2780 and SK-OV-3 and Chinese hamster ovary (CHO) cells (Figure S1A). Serum-free *in vitro* sphere formation assay showed that PRL-3 enhanced higher sphere efficiency than those of GFP parental cells, and the spheres induced by PRL-3-overexpressing cells were tighter than those in parental GFP cells (Figures 1A, 1B, and S1B). Moreover, ALDEFLUOR assay showed that aldehyde dehydrogenase (ALDH) activity, a stem-like character, is higher in PRL-3-overexpressing cells than in GFP cells under both adherent condition and the suspension transition state (Figure 1G). In contrast, knockdown of endogenous PRL-3 with specific short hairpin RNAs (shRNAs) in A2780 cells (Figure S1C) reduced the cell sphere formation efficiency (Figure 1C) and the ALDH activity in cells (Figure 1G). To exclude the possible effect of cell type on PRL-3 in enhancing cell sphere efficiency, we established an inducible PRL-3 expression system in CHO cells that have marginal endogenous PRL-3. With the increase of PRL-3 expression by doxycycline induction, the efficiency of cell sphere formation accordingly increased; however, when PRL-3 expression level reaches a threshold, the extra induced PRL-3 will not contribute to further cell sphere formation (Figure 1D). Immunofluorescence staining of Nanog, a key stem cell marker that functionally maintains cell stemness, demonstrated similar staining intensities of Nanog between the spheres induced by PRL-3-overexpressing cells and GFP parental cells (Figure 1E), indicating that when cell sphere is induced, there is no obvious phenotypical difference between the two types of spheres. To verify if there is renewal ability distinction between these two types of spheres, we performed serial passages of these spheres and ALDEFLUOR assay analysis of tumor spheres. Results showed that there was no clear difference in both renewal ability and sub-population percentage between the PRL-3-positive and the normal control spheres (Figures 1F and S1D). Thus, we concluded that PRL-3 might play an important role in the expansion of general tumor cells to CSCs, but not in the formed stem-like cells.

*In vivo* limiting dilution assay of tumor cells is considered as the gold standard to validate CSC stemness. Using this strategy, we observed that PRL-3 enhances tumorigenic efficiency of ovary tumor cells under normal adhesion culture condition at 10^4^ cells inoculation per mouse, compared with that of the parental cells. When we examined the tumorigenic efficacy of the cells dispersed from the formed spheres, we found that there was no discrepancy in xenografted tumor formation between the two types of the spheres at all the indicated cell number-diluted inoculations (Figure 1H). These results are further indicative of the role of PRL-3 in promoting stem-like tumor sphere formation under suspension culture induction, but no effect on the formed stem-like cells. All above-mentioned results indicated that PRL-3 expanded the CSC-like sub-population possibly by promoting the transition of general tumor cells to stem-like tumor cells.

### SOX2 Is an Indispensable Player in PRL-3-Enhanced CSC Transition

**Figure 2:**
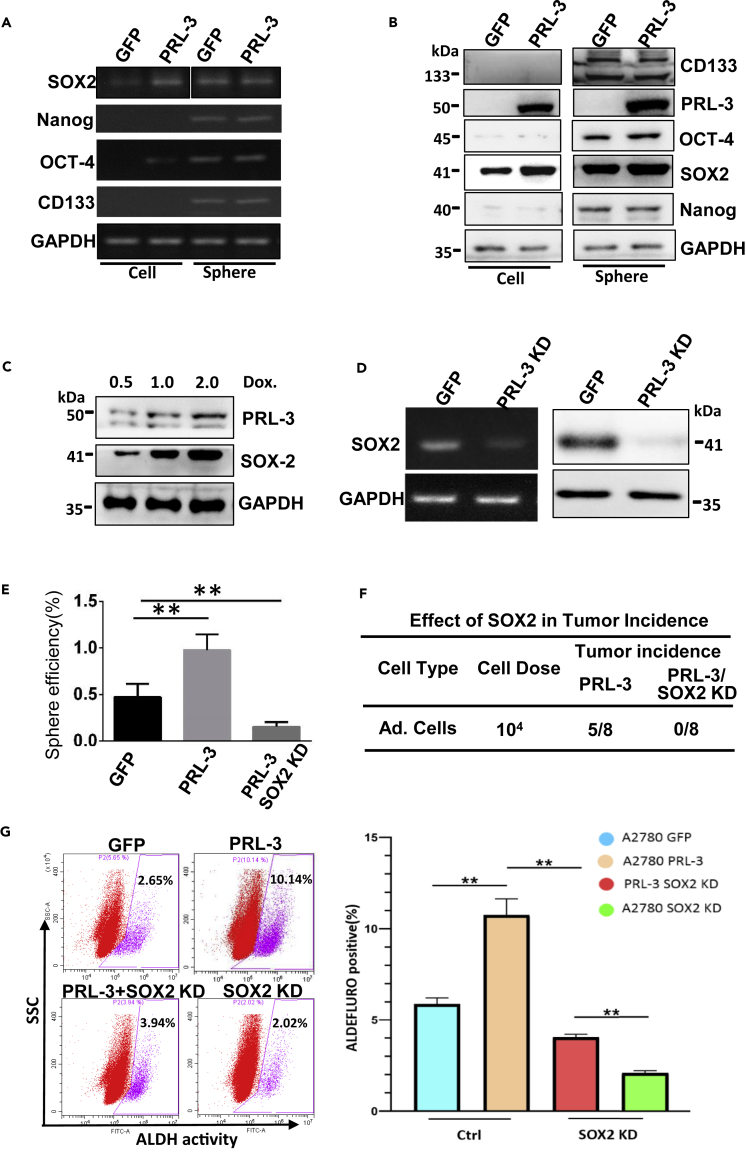
SOX2 Is an Indispensable Player in PRL-3-Enhanced CSC Transition (A) RT-PCR analyses of the indicated stem cell markers. Total RNA was isolated from A2780 GFP, A2780 GFP-PRL-3, A2780 GFP sphere, and A2780 PRL-3 sphere. Glyceraldehyde-3-phosphate dehydrogenase (GAPDH) was used as a loading control. (B) Immunoblots of the indicated stem cell markers in A2780 GFP, A2780 GFP-PRL-3, A2780 GFP sphere, and A2780 PRL-3 sphere with their specific antibodies. (C) Immunoblots of the inducible PRL-3 and SOX2 expressions in CHO cells with the indicated dose of doxycycline (Dox.) induction. Cells were transfected with a doxycycline-inducible PRL-3 expression plasmid to establish a stable cell line. (D) RT-PCR detection and immunoblot of Sox2 expression in PRL-3 knockdown cells. (E) Tumor spheres formed by A2780 GFP, A2780 PRL-3, and A2780 PRL-3 SOX2 KD cells were counted and sphere efficiency was calculated as previously described. (F) *In vivo* xenograft tumor formation comparison of A2780 PRL-3 cells with PRL-3 cells with additional Sox2 knockdown. (G) ALDEFLUOR assay of A2780 GFP, A2780 PRL-3, A2780 GFP SOX2 KD, and A2780 PRL-3 SOX2 KD cells. The fluorescent ALDH activity in the cells is measured by a CytoFLEX flow cytometer. The left panel shows the representative plots of the ALDH^br^ sub-population in the indicated cells, and the right panel shows the statistical analyses based on at least three independent experiments. Data are represented as mean ± SEM, ∗∗p < 0.01, unpaired *t* test.

To investigate how PRL-3 enhances the normal tumor cells to the stem-like cells, we first examined the general cell stemness markers. SOX2 and OCT-4 mRNA levels were increased in PRL-3-overexpressing cells in the normal culture condition (adhesion), but there was no obvious discrepancy between the formed stem-like spheres in terms of all the key stemness factors checked, including Nanog, OCT4, and CD133 (Figure 2A). Immunoblotting results further clearly confirmed that Sox2 protein level was exclusively upregulated in cells with PRL-3 overexpression under normal culture state, whereas in the spheres formed from the parental and PRL-3 cells, all stem markers showed similar expression levels, especially Sox2 (Figure 2B). The same results were observed from SK-OV-3 cells (Figures S2A and S2B). To check if PRL-3 works in a dose-dependent manner, the inducible PRL-3 expression CHO cell model was used again. Results showed that the increased PRL-3 level was accompanied by the increased Sox2 expression (Figure 2C), validating the above dose-dependent sphere formation efficiency with PRL-3 induction. In contrast, when the endogenous PRL-3 was knocked down by shRNAs (Figure S2C), Sox2 expression was reversely reduced on both mRNA and protein levels (Figures 2D, S2A, and S2B). Our results indicate that PRL-3 plays an important role in cell state transition of ovarian tumor cells to the cancer stem-like cells, possibly via SOX2 upregulation.

Given that Sox2 is one of crucial stemness driver in the induced pluripotent stem cells, we further unveiled if PRL-3 endows this cell-state transition via SOX2 upregulation effect. By additional knockdown of SOX2 in PRL-3-overexpressing cells (Figures S2D and S2F), tumor sphere induction assay and ALDEFLUOR assay showed that silencing of SOX2 almost blocks PRL-3's effect (Figures 2E and 2G). *In vivo* tumor formation assay also confirmed this effect of SOX2 that mediates PRL-3's function in stem-like tumor sphere formation (Figure 2F), indicating that SOX2 is a possible master player in PRL-3-induced expansion of CSC sub-population.

### PRL-3 Increases MEF2A Binding to SOX2 Promoter for SOX2 Upregulation

**Figure 3:**
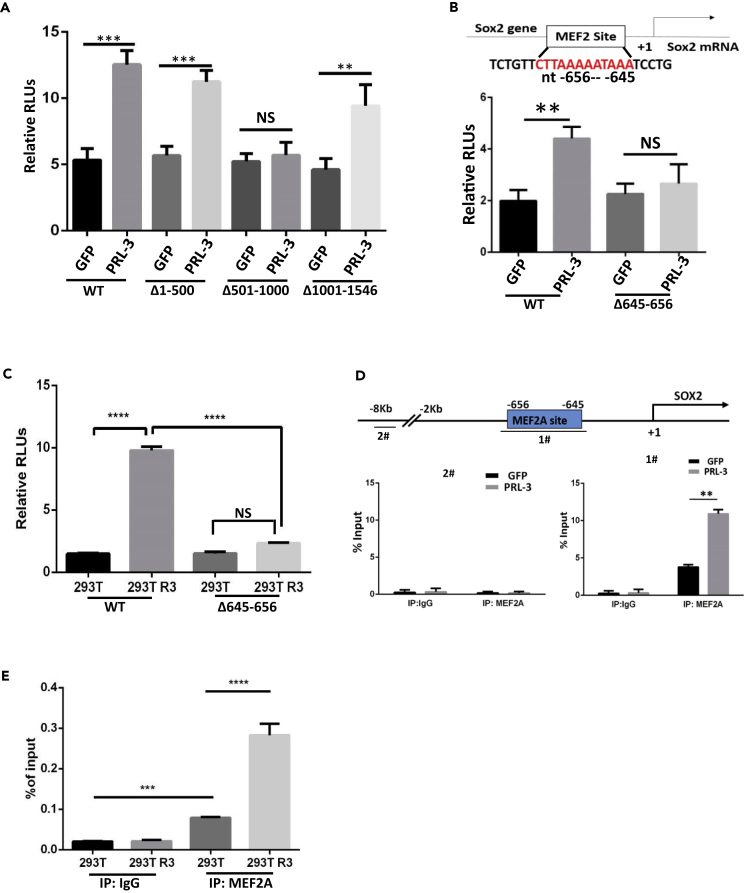
PRL-3 Increases MEF2A Binding to SOX2 Promoter for SOX2 Transcriptional Upregulation (A) Luciferase reporter analysis of SOX2 transcription in both A2780 GFP and A2780 PRL-3 cells parallelly. The indicated full-length SOX2 promoter construct (WT) and its serial deletion mutants (Δ) were transfected into cells. After 48 h, the luciferase activities were robustly measured as described in Methods. The secreted alkaline phosphatase (SEAP) was used as an internal control to normalize the results. All experiments were triply performed. Data are represented as mean ± SEM, ∗∗∗p < 0.001, ∗∗p < 0.01, unpaired *t* test. (B) Luciferase reporter assay of the full-length (WT) and a deletion mutant (Δ645-656) of SOX2 promoters as in (A). Nucleotides (nt) 645–656 is a predicted crucial MEF2A-binding sequence. ∗∗p <0.01, unpaired *t* test. (C) Luciferase reporter assay of the full-length (WT) and a deletion mutant (Δ645–656) of SOX2 promoters in 293T or 293T PRL-3 cells, assayed as in (B). ∗∗∗∗p < 0.0001, unpaired *t* test. (D) Chromatin immunoprecipitation of MEF2A-bound SOX2 promoter fragments with specific MEF2A antibody in both A2780 GFP and A2780 PRL-3 cells. IgG was used as a negative control. The bound Sox2 promoter fragment flanking nt-645–656 (fragment #1) was examined by quantitative PCR. The 8-kb far upstream fragment (fragment #2) was used as a negative control. Data are represented as mean ± SEM, ∗∗p < 0.01, unpaired *t* test. (E) Chromatin immunoprecipitation of MEF2A-bound SOX2 promoter fragment (fragment #1) in both A2780 GFP and PRL-3 cells as in (D), ∗∗∗∗p < 0.0001, ∗∗∗p < 0.001, unpaired *t* test.

As Sox2 is transcriptionally regulated on multiple levels by various effectors, including *cis*-elements, enhancers, transcription factors, microRNA (miRNAs), or long non-coding RNAs (Wiebe et al., 2000, Tomioka et al., 2002, Zhou et al., 2014, Jia et al., 2012, Rutenberg-Schoenberg et al., 2016), to characterize how SOX2 is upregulated by PRL-3, we first carried out a luciferase assay. We fused SOX2 promoter sequence (nt +226 to −1320) with luciferase-encoding gene, and results showed that PRL-3 overexpression evidently increased luciferase activity, manifesting a transcriptional regulation of Sox2 expression (Figure 3A-WT; Figure S3A). Serial deletions of the SOX2 promoter region showed a potential fragment (nt-501–1000 bp) that may be responsible for the PRL-3-induced luciferase activity or Sox2 expression (Figure 3A, Δ501–1000). With bioinformatic analysis of this region, we predicted transcription factor MEF2A with the highest binding probability in this SOX2 promoter region, especially in the nt-645–656 portion (Figure S3B). As only MEF2A and MEF2D were indeed detected in A2780 cells, we first knocked down transcription factor MEF2D and found no effect on Sox2 expression (Figure S3C), hinting the possible MEF2A effect here. Deletion of MEF2A-binding site nt-645–656 portion almost mimicked the deletion of nt-501–1000 bp, suggesting that MEF2A is indispensable for PRL-3-induced Sox2 expression (Figure 3B). To exclude the tumor cell context effect of SOX2 expression, we also conduct this luciferase reporter assay in 293T cells and found similar crucial role of nt-645–656 for SOX2 expression (Figure 3C), suggesting general regulation of SOX2.

On further knockdown of MEF2A with two specific small interfering RNAs, we observed a decrease in PRL-3-induced Sox2 expression (Figures S3D and S3E). Similarly, after overexpressing MEF2A protein (Figure S3F), an obvious increase in Sox2 expression was detected in both parental control and PRL-3-overexpressing cells, but higher SOX2 level still existed in PRL-3 cells (Figure S3G). More importantly, chromatin immunoprecipitation (ChIP) with MEF2A antibody precipitated more enriched Sox2 promoter fragments flanking nt-645–656 portion in PRL-3 cells, compared with GFP parental cells (Figure 3D, fragment #1). The same results were obtained from normal 293T cells with chromatin immunoprecipitation (Figure 3E), indicating that MEF2A did bind to SOX2 promoter region, especially in the nt-645–656 region, to transcriptionally upregulate SOX2 expression.

### HDAC4 Bridges the Role of PRL-3 in MEF2A-Triggered SOX2 Transcription

**Figure 4:**
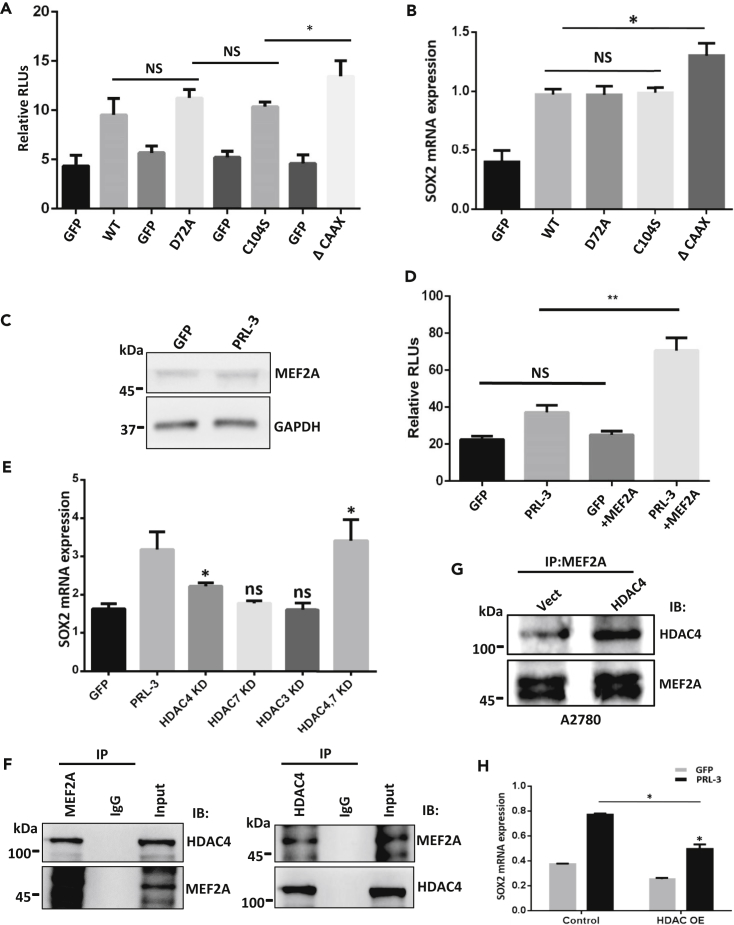
HDAC4 Mediates PRL-3 with MEF2A for SOX2 Transcription (A) Luciferase reporter assays of SOX2 promoter activity affected by PRL-3 (WT) or PRL-3 mutants. PRL-3 mutants include D72A (ATP-binding inactive), C104S (catalytically inactive), and ΔCAAX (cell membrane prenylation motif deletion, leading to the cytosol translocation). All experiments were conducted three times independently. Data are represented as mean ± SEM, ∗p < 0.05, unpaired *t* test. (B) Quantitative RT-PCR detection of Sox2 mRNA expression levels influenced by PRL-3 (WT) or PRL-3 mutants as in (A) in A2780 cells. Data are represented as mean ± SEM, ∗p < 0.05, unpaired *t* test. (C) Immunoblot of MEF2A protein levels in A2780 and PRL-3-overexpressing cells. GAPDH was used as a loading control. (D) Luciferase reporter assay of SOX2 promoter activity affected by MEF2A overexpression. All measurements were conducted three times independently. Data are represented as mean ± SEM, ∗∗p < 0.01, unpaired *t* test. (E) Quantitative RT-PCR detection of SOX2 transcripts upon knockdown (KD) of HDAC3, HDAC4, and HDAC7 with their small interfering RNAs. Data are represented as mean ± SEM, ∗p < 0.05, unpaired *t* test. (F) Co-immunoprecipitation (IP) of HDAC4 or MEF2A with respective MEF2A or HDAC4 antibody in HEK293T cells to validate the interaction between MEF2A and HDAC4. Cells were transfected with both pCGN-MEF2A and pcDNA-HDAC4-FLAG vectors. (G) Immunoprecipitation of HDAC4 with MEF2A antibody in A2780 cells with additional HDAC4 overexpression. (H) Quantitative RT-PCR examination of SOX2 transcription in A2780 GFP and PRL-3 cells, after forced HDAC4 expression. Data are represented as mean ± SEM, ∗p < 0.05, unpaired *t* test.

The metastatic role of PRL-3 is often linked to its function as a tyrosine phosphatase (Saha et al., 2001, Alonso et al., 2004, Guo et al., 2004, Kim et al., 2004). Therefore, we sought to know whether PRL-3's phosphatase function participates in Sox2 upregulation. Both sphere formation and luciferase reporter assays showed that catalytically inactive PRL-3 (D72A and C104S) exerted the same enhancive effects on Sox2 promoter activity and sphere formation efficiency as the wild-type PRL-3 (Figures 4A and S4A), indicating the phosphatase-independent function of PRL-3 in CSC-like transition. Meanwhile, a mutant with deletion of native PRL-3 prenylation motif (ΔCAAX), from which PRL-3 loses its inner cell membrane localization, enhanced the luciferase activity and tumor sphere formation (Figures 4A and S4A). Likely, Sox2 mRNA levels remained the same in the phosphatase-inactive mutants as that of wild-type PRL-3, but increased in the prenylation-defect mutant (Figure 4B), suggesting a possibility that it is the cytoplasmic translocation of PRL-3 that enhances Sox2 expression, rather than its phosphatase activity. We detected the MEF2A level and observed an unexpected result that PRL-3 overexpression had no influence on MEF2A protein level (Figure 4C), but MEF2A overexpression indeed could evidently further increase the luciferase activity in PRL-3-overexpressing cells, compared with the parental cells (Figure 4D). These results prompted us to suspect that PRL-3 may work somehow in an indirect manner on SOX2 expression by MEF2A.

The accessibility of MEF2 transcription factors to their target genes promoters for gene expression can be contributed by the nuclear export of Class IIa histone deacetylases (HDACs) to cause chromatin relaxation (Lu et al., 2000, Smith et al., 2007). HDAC4 has been shown to bind MEF2A directly to repress its transcription activity by the deacetylation modulation (Miska et al., 1999, Clocchiatti et al., 2013, Di Giorgio and Brancolini, 2016), and the nuclear imports of HDACs are phosphorylation regulated by phosphatase (Martin et al., 2008, Paroni et al., 2008). To clarify this possibility, we first straightforwardly silenced HDAC3, HDAC4, and HDAC7 in A2780 cells (Figure S4B). Depletion of HDAC4 could almost mimic the effect of PRL-3 overexpression, although HDAC7 has a somewhat similar influence, simultaneous knockdown of HDAC4 and HDAC7 led to a significant higher SOX2 expression, hinting that Class II deacetylases may be involved in this process in a redundant manner (Figure 4E). Therefore, HDAC4 was chosen as the key factor for the following study.

To check if there is a physical interaction between HDAC4 and MEF2A, HEK293T cells were co-transfected with both MEF2A and HDAC4-FLAG for co-immunoprecipitation analysis. Mutual immunoprecipitation with either MEF2A or HDAC4 antibody clearly validated the true interaction between MEF2A and HDAC4 (Figure 4F). Immunoprecipitation also showed that HDAC4 overexpression (Figure S4C) increased the MEF2A-bound HDAC4 amount in A2780 cells (Figure 4G). Overexpressing HDAC4 decreased SOX2 transcription in PRL-3-overexpressing cells, further confirming this notion (Figure 4H). This result was also confirmed by luciferase reporter assay (Figure S4D).

Together, the results here manifested that Class II HDAC4 played a mediating role in PRL-3-induced SOX2 expression through the interaction between MEF2A and HDACs.

### HDAC4 Coordinately Deacetylates MEF2A and Renders Histones H3 and H4 Relaxation to Promote MEF2A Accessibility to SOX2 Promoter

**Figure 5:**
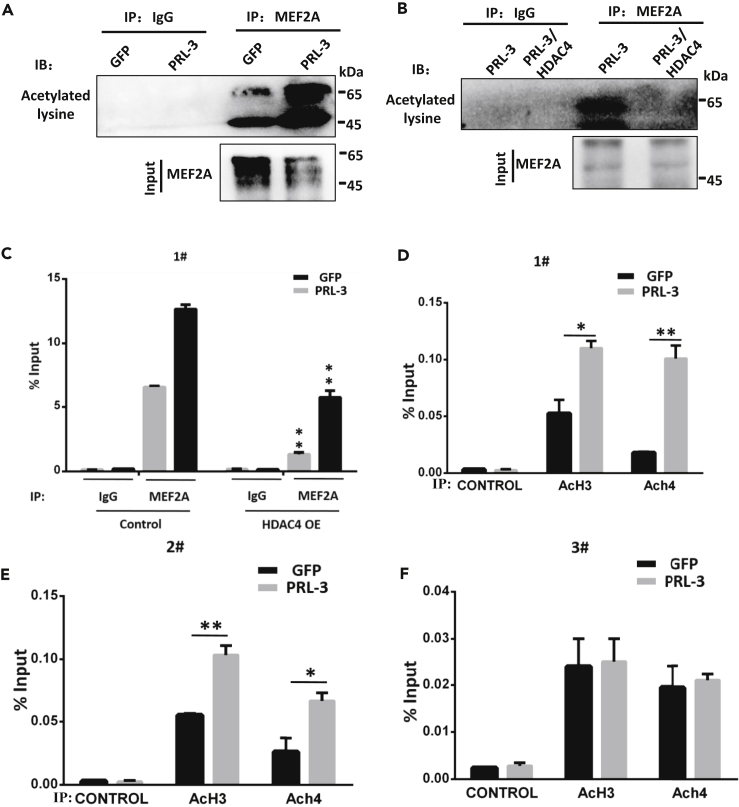
HDAC4 Concurrently Deacetylates MEF2A and Histones H3 and H4 to Facilitate MEF2A Binding to SOX2 Promoter (A) Immunoprecipitation (IP) detection of the acetylated MEF2A in A2780 GFP or A2780 GFP-PRL-3 cells. MEF2A acetylation was examined by immunoblot of the immunoprecipitated MEF2A with an anti-acetylated lysine antibody. (B) Immunoprecipitation detection of the acetylated MEF2A in A2780 GFP-PRL-3 cells with or without additional HDAC4 overexpression as in (A). (C) Chromatin immunoprecipitation of MEF2A-bound SOX2 promoter region in both A2780 GFP and PRL-3 cells with or without HDAC4 overexpression (OE), respectively, as before. MEF2A-bound Sox2 promoter fragment (fragment #1) was valued by quantitative PCR. (D–F) Histone acetylation status of promoter region examined with chromatin immunoprecipitated fragments of Sox2 promoter region in both A2780 GFP and PRL-3 cells by acetylated histone 3 (AcH3), or histone 4 (AcH4), in both A2780 GFP and PRL-3 cells as in (C). (D) MEF2A-bound fragment (fragment #1) precipitated. (E) MEF2A flanking region (fragment #2) precipitated. (F) The 8-kb far upstream fragment (fragment #3) precipitated showed no difference in histone acetylation. Data are represented as mean ± SEM, ∗∗p < 0.01, ∗p < 0.05, unpaired *t* test.

Class II HDAC, including HDAC4, functioning as an instinct deacetylase, can remove MEF2A-acetylated groups upon their interaction, whereas the disassociation of HDAC4 from MEF2A inversely could increase MEF2A acetylation, leading to active transcriptional events of target genes (Spange et al., 2009, Joseph et al., 2017, Yuan et al., 2014, Smith et al., 2008, Ishikawa et al., 2010). To clarify whether HDAC4 can modulate MEF2A acetylation in SOX2 expression regulation event, we first checked MEF2A acetylation status. Immunoprecipitating with MEF2A and checking with the acetylated lysine antibodies revealed a pronounced acetylated MEF2A level in PRL-3 cells (Figure 5A), indicating the positive modulation of MEF2A acetylation by PRL-3. Once HDAC4 overexpression was committed in PRL-3 cells, MEF2A acetylation state was clearly decreased (Figure 5B), verifying the blockade effect of HDAC4 on PRL-3-mediated MEF2A acetylation. We then conducted additional ChIP analysis and demonstrated that the additional HDAC4 overexpression in PRL-3 cells suppressed MEF2A binding to SOX2 promoter region (fragment #1) (Figure 5C), verifying the favorable effect of MEF2A acetylation on its accessibility to SOX2 promoter.

HDAC4 not only deacetylates transcription factors but also promotes gene transcription through the coordinated histone deacetylation for chromosome remodeling in another side, especially via H3 and H4 deacetylations (Groth et al., 2007, Shahbazian and Grunstein, 2007, Tse et al., 1998, Wang et al., 2001, Shogren-Knaak et al., 2006, Davie et al., 2008). Therefore, alteration of HDACs would collectively cause universal chromatin relaxation for transcription factors' entry and binding. To confirm this possibility, we performed ChIP analysis on MEF2A binding to SOX2 promoter region again. ChIP results confirmed that there was increased amount of the acetylated histone H3 and H4 linked with the MEF2A binding fragments in Sox2 promoter regions (Figure 5D, fragments #1; 5E #2 fragments), whereas the far upstream regions could not be precipitated by the acetylated histones H3 and H4 (Figure 5F, fragment #3). Either HDAC4 knockdown or its overexpression indeed significantly increased or decreased the precipitation of MEF2A fragments with acetylated histones H3 and H4, respectively, indicating the negative effect of HDAC4 on gene transcription via chromatin remodeling (Figure S5F).

Altogether, our results supported the suspect that HDAC4 represses MEF2A-dependent transcription of SOX2 and the deacetylations of MEF2A and histones H3 and H4, which may render effective MEF2A access and binding to Sox2 promoter region.

### PRL-3 Interacts with and Attracts HDAC4 Translocation to Degradation

**Figure 6:**
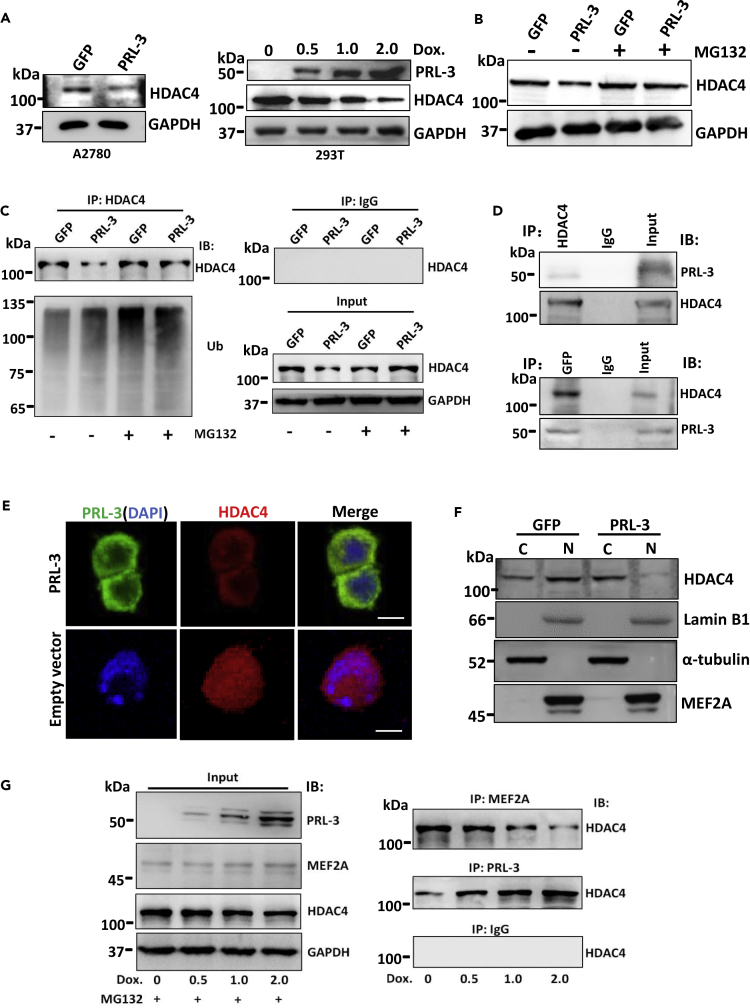
PRL-3 Interacts with HDAC4 to Attract HDAC4 Translocation and Degradation (A) Immunoblots of HDAC4 in both A2780 and PRL-3-inducible 293T cells. (B) Immunoblots of HDAC4 in A2780 GFP and A2780 PRL-3 cells with or without MG 132 treatments. (C) Ubiquitination of HDAC4 affected by PRL-3. Immunoprecipitation (IP) of HDAC4 in both A2780 GFP and A2780 GFP-PRL-3 cells with HDAC4 antibody, and detected with ubiquitin antibody by immunoblotting (IB). Cells treated with or without MG 132. (D) Mutual immunoprecipitation (IP) of PRL-3 and HDAC4 in A2780-GFP-PRL-3 cells and examined by the indicated antibodies (immunoblotting [IB]). (E) Immunofluorescence staining of HDAC4 in A2780 GFP and A2780 GFP-PRL-3 cells. Nuclei were stained with DAPI. Scale bars, 10 μm. (F) Sub-cellular fractions and immunoblots of HDAC4 and MEF2A in A2780 GFP and A2780 GFP-PRL-3 cells. Nuclear (N) and cytoplasmic (C) proteins are indicated. Lamin B1 and α-actin were used as nuclear and cytoplasmic protein markers, respectively. (G) IP of HDAC4 with either MEF2A or PRL-3 antibody in PRL-3-inducible expression 293T cells with MG 132 pre-treatment to inhibit HDAC4 degradation. Binding of HDAC4 to MEF2A or PRL-3 was examined by IB.

Considering the above-mentioned results that PRL-3 phosphatase activity was not involved in SOX2 expression, but the prenylation defect PRL-3 mutant was (Figures 4A and S4A), as well as that PRL-3 increased MEF2A acetylation (Figure 5A), we speculated the probability that PRL-3 and HDAC4 might compete with each other to bind with MEF2A through their interaction. To confirm the hypothesis, we first tentatively detected HDAC4 expression and found that HDAC4 was reduced in PRL-3-overexpressing A2780 cells (Figure 6A). Furthermore, in the inducible PRL-3 system, we also observed that HDAC4 level decreased clearly, along with PRL-3 level increase (Figure 6A), indicating that PRL-3 downregulates HDAC4 expression. When treating the cells with the proteasome inhibitor, MG132, the reduced HDAC4 levels by PRL-3 overexpression were restored back to those in various parental cells (Figure 6B), hinting that PRL-3 exerted the proteasome-dependent HDAC4 degradation. Ubiquitination analysis really demonstrated the heavily ubiquitinated HDAC4 in PRL-3 overexpressing cells (Figure 6C).

Class II HDAC4 is known to shuttle between the cytoplasm and nucleus to quickly respond to environment change of cells (Nishino et al., 2008, Grozinger and Schreiber, 2000). Given that HDAC4 actively exports from the nucleus to cytoplasm (Miska et al., 1999, Borghi et al., 2001, Di Giorgio and Brancolini, 2016), there are very high chances for interaction between the cytosolic PRL-3 and HDAC4. We sought to check whether this sort of interaction exists; we performed mutual immunoprecipitations and found that PRL-3 and HDAC4 were really precipitated by each other (Figure 6D), although relatively small amount of PRL-3 was precipitated with respect to the total input, which might be due to the low level of endogenous HDAC4 protein in the cells. Immunofluorescence staining also showed that PRL-3 overexpression indeed co-localized with HDAC4 in the cytosol; in contrast, HDAC4 is located in the nuclei of PRL-3-null cells (Figure 6E), suggesting that PRL-3 overexpression attracts HDAC4 export from the nucleus to cytoplasm. To confirm this translocation, we sub-fractioned cytoplasmic and nuclear contents of the cells, and immunoblotting detection showed much more HDAC4 in the nuclei of PRL-3-null cells, whereas the majority of HDAC4 in the cytoplasm of PRL-3-overexpressing cells (Figure 6F). These results are consistent with the previously reported ones in HeLa cells (Miska et al., 1999).

Upon inhibition of HDAC4 proteosome degradation with MG132, we observed a decreased interaction between HDAC4 and MEF2A in PRL-3-overexpressing cells, compared with the parental cells (Figure S6B). In addition, in the inducible PRL-3 expression system, we also found that with the increasing PRL-3 amounts, increasing amounts of HDAC4 bound to PRL-3; in contrast, less HDAC4 bound to MEF2A upon MG132 treatment to stop HDAC4 degradation (Figure 6G). Similarly, the decreasing amount of HDAC4 bound to MEF2A along with the increasing amounts of PRL-3 induction was also confirmed in CHO cells (Figure S6C), and more HDAC4 bound to the increased PRL-3 once HDAC4 degradation was inhibited (Figure S6D), validating the dissociation of HDAC4 from MEF2A to bind to PRL-3 for the eventual ubiquitination-mediated degradation.

Taken together, results here demonstrated that PRL-3 overexpression could attract HDAC4 to translocate from the nuclei to cytoplasm for their interaction and the subsequent proteasome degradation, leading to the release of individually acetylated MEF2A that can bind onto Sox2 promoter region.

### Relevance of PRL-3, SOX2, and HDAC4 in Clinical Ovarian Tumors

**Figure 7:**
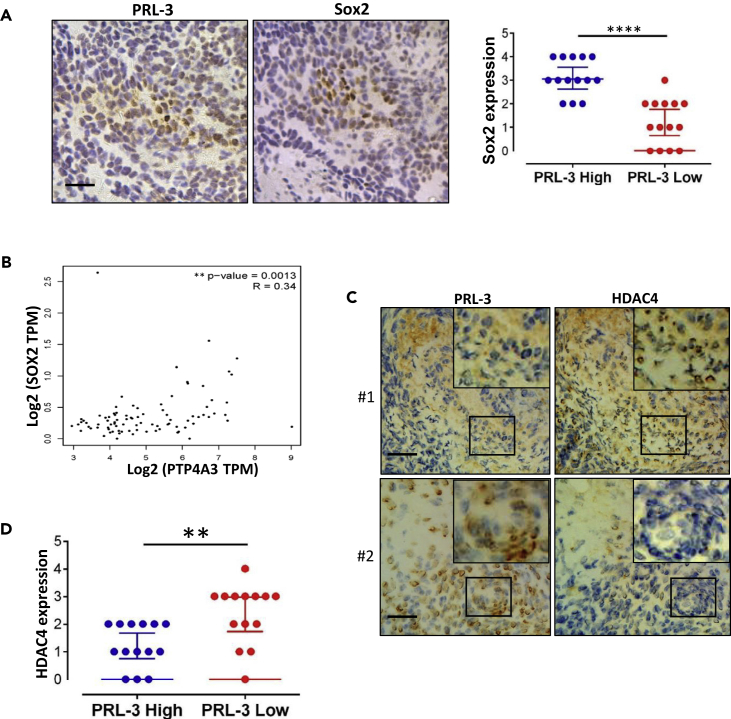
Relevance of PRL-3, SOX2, and HDAC4 in Clinical Ovarian Tumors (A) Immunohistochemistry analyses of PRL-3 and Sox2 expressions in 28 freshly collected ovarian cancer samples. The statistical expression of SOX2 in PRL-3-high and PRL-3-low groups is clustered, Data are represented as mean ± SEM, ∗∗∗∗p < 0.0001, unpaired *t* test. Scale bar, 50 μm. (B) Correlationship between PRL-3 and SOX2 expression in clinical data. The Cancer Genome Atlas database of ovary cancer was analyzed with GEPIA, ∗∗p < 0.01, Spearman test. (C) Immunohistochemistry analyses of HDAC4 and PRL-3 expressions in ovarian cancer samples by serial sections in (A). Inserts showing the amplified regions to show the opposite expression pattern of PRL-3 to HDAC4 in the same cells. #1 Scale bar, 100 μm, #2 scale bar, 50 μm. (D) The statistical analysis of HADC4 expression in PRL-3-high and PRL-3-low groups, Data are represented as mean ± SEM, ∗∗p < 0.01, unpaired *t* test.

To validate if PRL-3-high tumors really have high level of SOX2 in clinical samples, we collected and examined both PRL-3 and Sox2 expressions in 37 fresh ovarian cancer samples. All samples were classified into PRL-3-low (with score 0, 1, 2) and PRL-3-high (with score 3, 4) groups, based on the robust immunohistochemical (IHC) staining intensity. IHC analysis showed not only that SOX2 expression was significantly higher in the PRL-3-high group than in the PRL-3-low group but also that both expression patters were well co-localized (upper panel, a pair of serially sliced samples) and there was a tight correlation between PRL-3 and SOX2 expression in these tumor samples (Figure 7A). In addition, The Cancer Genome Atlas datasets of ovarian cancer clinical samples analysis validated a good correlation between PRL-3 and SOX2 expressions at mRNA levels (Figure 7B, n = 295).

Moreover, we analyzed HDAC4 expression in the PRL-3-low and PRL-3-high fresh ovarian cancer groups. IHC results also manifested that HDAC4 expression was inversely correlated with PRL-3 in these tumor samples (Figures 7C and 7D). This information further confirmed the real bridge role of HDAC4 in PRL-3-induced SOX2 expression.

## Discussions

Stem-like cancer cells are recognized as a small subset of cells that has the ability to repopulate multiple tumors in different sites. EMT and drug resistance are believed to be the key characters of these CSCs. We have revealed that PRL-3 can induce EMT through PTEN downregulation (Wang et al., 2007) and can lead to drug resistance of AML, implying the possibility of PRL-3 in tumor stem-like cell transition. Here we experimentally demonstrated that PRL-3 can promote the expansion of CSC sub-population via the coordinated regulations of both acetylation states of a transcription factor MEF2A for the key stemness factor SOX2 transcriptional expression and histones for chromatin relaxation. In this process, PRL-3 binds to HDAC4 to render its translocation and degradation, leading to both acetylated MEF2A and histones for effective MEF2A binding to Sox 2 promoter region (Figure 7E). Thus, we here disclosed a mechanistic understanding of cancer relapse, which extends the metastatic PRL-3 to be a cancer relapse driver. Cancer stem-like cells have been recognized to be critical for cancer dormancy upon chemotherapies, and now they are the main targets for developing second-line therapeutic methods, due to their abilities in self-renewal, tumor initiation, metastasis, and relapse (Clevers, 2011). Ovarian cancer cells with CD44+/CD24-, CD133, or ALDH show higher likelihood of recurrence, resistance to standard chemotherapy and radiotherapy, and poor prognosis (Meng et al., 2012, Zhang et al., 2012, Stemberger-Papic et al., 2015, Landen et al., 2010). In line with this, PRL-3 overexpression results in more ALDH-positive sub-population, indicating that patients with higher PRL-3 level may tend to develop drug resistance and recurrence. Thus, we propose PRL-3 as a promising therapeutic target for thorough eradication of dormant cancer cells.

PRL-3 is known as a tyrosine phosphatase linked to metastasis of various cancers, strictly dependent on its phosphatase activity (Al-Aidaroos and Zeng, 2010). However, the specific substrate of PRL-3 is still elusive by far, hinting the possible multifunction of PRL-3 in cancer progression. Our results here showed that PRL-3 works as an adaptor, which is independent of its phosphatase activity, to promote expansion of CSC sub-population possibly via the transition from normal ovarian cancer cell to the stem-like cancer cells. In this process, the cellular localization of PRL-3 indeed is critical (Figures 4A and 4B), enlightening an important consideration of a key protein's location with its specific function in cancers or other diseases, for instance, of p27 and p53, whose locations are linked to various outcomes in cancer progression (Larrea et al., 2009, Muller and Vousden, 2014). Therefore, the non-enzymatic manner of such key phosphatases should not be ignored in a particular scenario. PRL-3 was found in the nucleus of colorectal cancer cells (Liu et al., 2013b) and can promote telomere deprotection to maintain chromosomal instability (Lian et al., 2017). Our results currently further show that PRL-3 independent of its phosphatase activity binds to the deacetylase HDAC4, in the cytosol, to attract the disassociation of HDAC4 from MEF2A to export to cytoplasm, leading to the consequent HDAC4 degradation. The underlying mechanism of how PRL-3 attracts and brings HDAC4 export from nucleus, and how HDAC4 undergoes ubiquitination degradation, needs to be further investigated. Tentatively, we proposed a possibility that when the cytoplasmic PRL-3 increased to a certain level, it would attract the dynamic HDAC4 from the nucleus like a sponge or directly enter into the nucleus to bind to MEF2A with higher affinity, resulting in a one-way shuttle of HDAC4 to the cytosol for ubiquitination and degradation (Figure 6).

Acetylation and deacetylation modifications, as main epigenetic regulations, play mutual balances in cell physiology and homeostasis. HDAC4 is one of the key deacetylases to modulate both transcription factors and histones, leading to a large event of cell differentiation or differentiation in cell fate (Di Giorgio and Brancolini, 2016). Here we unexpectedly observed that this small phosphatase PRL-3 even could participate in such complex process, especially in the modulation of the transcription of a stem-cell factor, SOX2, based on the acetylation status of its transcription factor, MEF2A, and the corresponding chromatin locus accessibility for MEF2A entry and binding. Other concomitant events should be checked to indicate the specific physiological effect of PRL-3 and HDAC4 in the cancer cell state transition. Thereafter, PRL-3 itself again may be considered as a potential therapeutic target in diseases due to epigenetic abnormal regulation, including various types of cancer, rather than HDAC4 that would raise generally intrinsic side effect if targeted.

SOX2 is aberrantly expressed in various cancers (Liu et al., 2013a). Given its key stemness factor character, the Sox2 expression regulation is necessary to be figured out. Besides basal promoter and distal enhancers, miRNAs and long non-coding RNAs are also involved in the transcriptional control of Sox2 expression (Wiebe et al., 2000, Zappone et al., 2000, Tomioka et al., 2002, Miyagi et al., 2006, Leis et al., 2011, Zhou et al., 2014). In this study, we found PRL-3 working as an upstream effector to upregulate Sox2 at the transcriptional level. This study shows that the influence of PRL-3 alone on stemness is limited; only when cells are cultured in non-adherent and serum-free conditions it promotes the numbers of spheres, which is consistent with the observed fact that PRL-3 is often detected in metastatic sites. However, the other key factors, including Nanog, OCT4, or CD133, have not been impaired, to reason the accelerated transition of stem-like tumor cell formation, but not the formed tumor stems cells. Likewise, others studies have shown that Sox2 expression plays key role in sphere formation efficiency (Rodriguez-Pinilla et al., 2007, Hagerstrand et al., 2011, Wu et al., 2012, Rita et al., 2009, Basu-Roy et al., 2011, Bourguignon et al., 2012, Leis et al., 2011, Singh et al., 2012). Thus, PRL-3 could be a potential player in normal tissue stem cell homeostasis, for instance, in the expansion of pluripotent stem cell population. To bypass the potentially undesired effect of SOX2-targeting therapy, PRL-3 could be alternatively focused on. Interestingly, the continuous elevation of PRL-3 can induce increased Sox2 expression, but there is no further sphere efficiency increase, indicating a threshold amount of Sox2 in stem-like cell transition.

### Limitations of the Study

• The expansion of CSC-like sub-population in PRL-3-positive cells could be due to other mechanisms, such as the blocked differentiation or a more active stem cell division.
• Given the general effect of histone acetylation, other factors may also be modulated when HDAC4 undergoes translocation or degradation.
• The sample size of patients with ovarian cancer is relatively small.

## Methods

All methods can be found in the accompanying Transparent Methods supplemental file.

## Supplemental Information

Document S1. Transparent Methods, Figures S1–S6, and Table S1
