# Supplementary material for: Metastatic Phosphatase PRL-3 Induces Ovarian Cancer Stem Cell Sub-population through Phosphatase-Independent Deacetylation Modulations
Source: iScience. 2019 Dec 12;23(1):100766. doi: 10.1016/j.isci.2019.100766 (PMC6941878; doi:10.1016/j.isci.2019.100766)
Supplement: Document S1. Transparent Methods, Figures S1–S6, and Table S1 [file mmc1.pdf]

ISCI, Volume *23*

Supplemental Information

Metastatic Phosphatase PRL-3 Induces Ovarian Cancer Stem Cell Sub-population through Phosphatase-Independent Deacetylation Modulations

Mingming Zhang, Yanli Wei, Yanbin Liu, Wen Guan, Xiaomei Zhang, Jianqiu Kong, Hui Li, Shulan Yang, and Haihe Wang

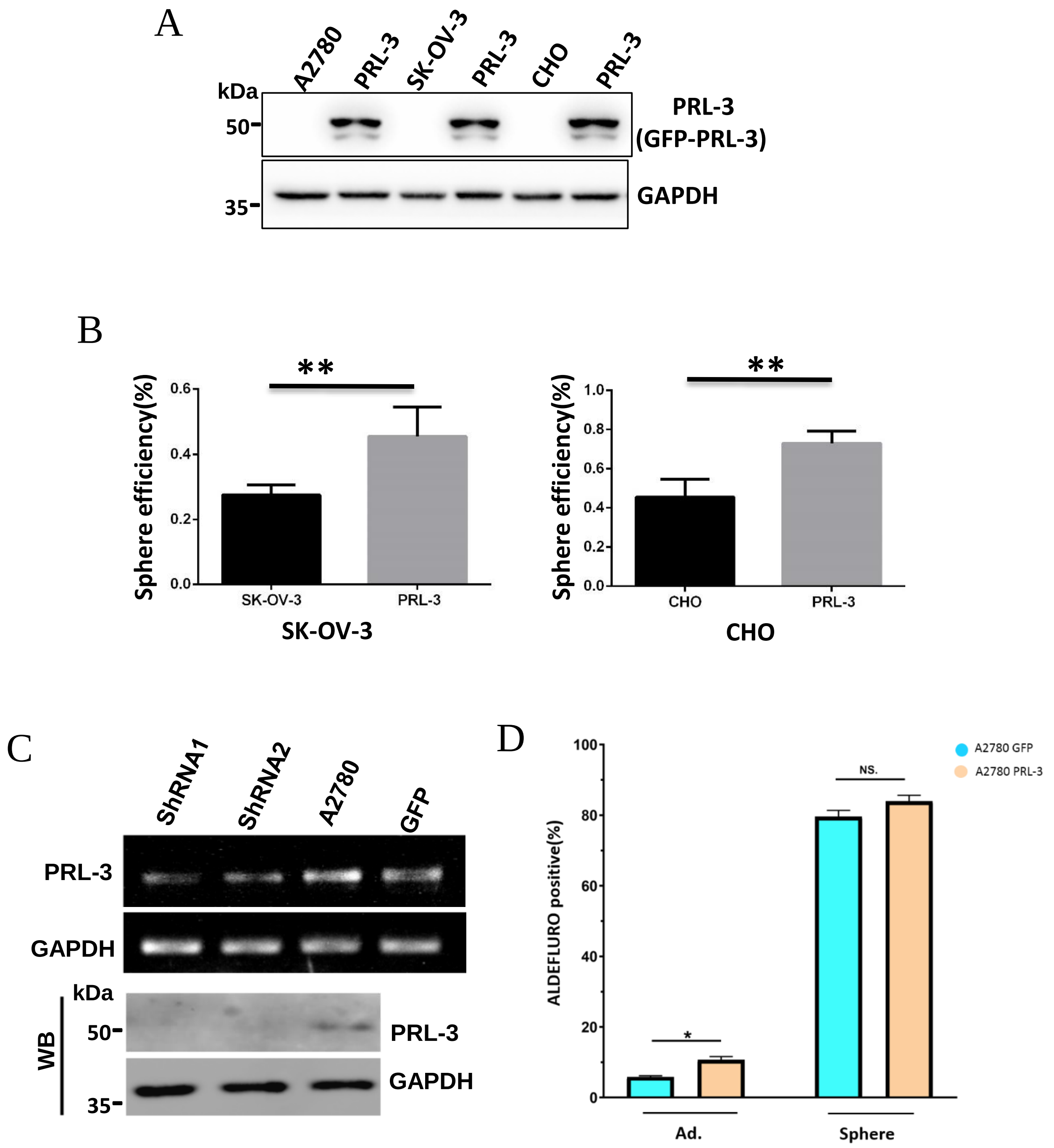

Figure S1. PRL-3 promotes cancer stem-like spheres, Related to Figure 1

A. Immunoblots of GFP-PRL-3 in A2780, SK-OV-3 and CHO cells.

B. Sphere formation efficiencies of SK-OV-3 and CHO cells transfected with GFP empty vector or GFP-PRL-3. After 3-5 days of seeding, efficiencies were calculated as described in Transparent Methods. Data are represented as mean ±SEM, **p<0.01, unpaired *t*-test.

C. Immunoblots and RT-PCR of endogenous human PRL-3 after knocking down endogenous PRL-3 (KD), compared to the wild type cells or cells transfected with GFP vector.

D. Aldefluor assay of A2780 or A2780 PRL-3 cells under adherent culture condition or after sphere formation. Data are represented as mean ±SEM, *p<0.05, unpaired *t*-test.

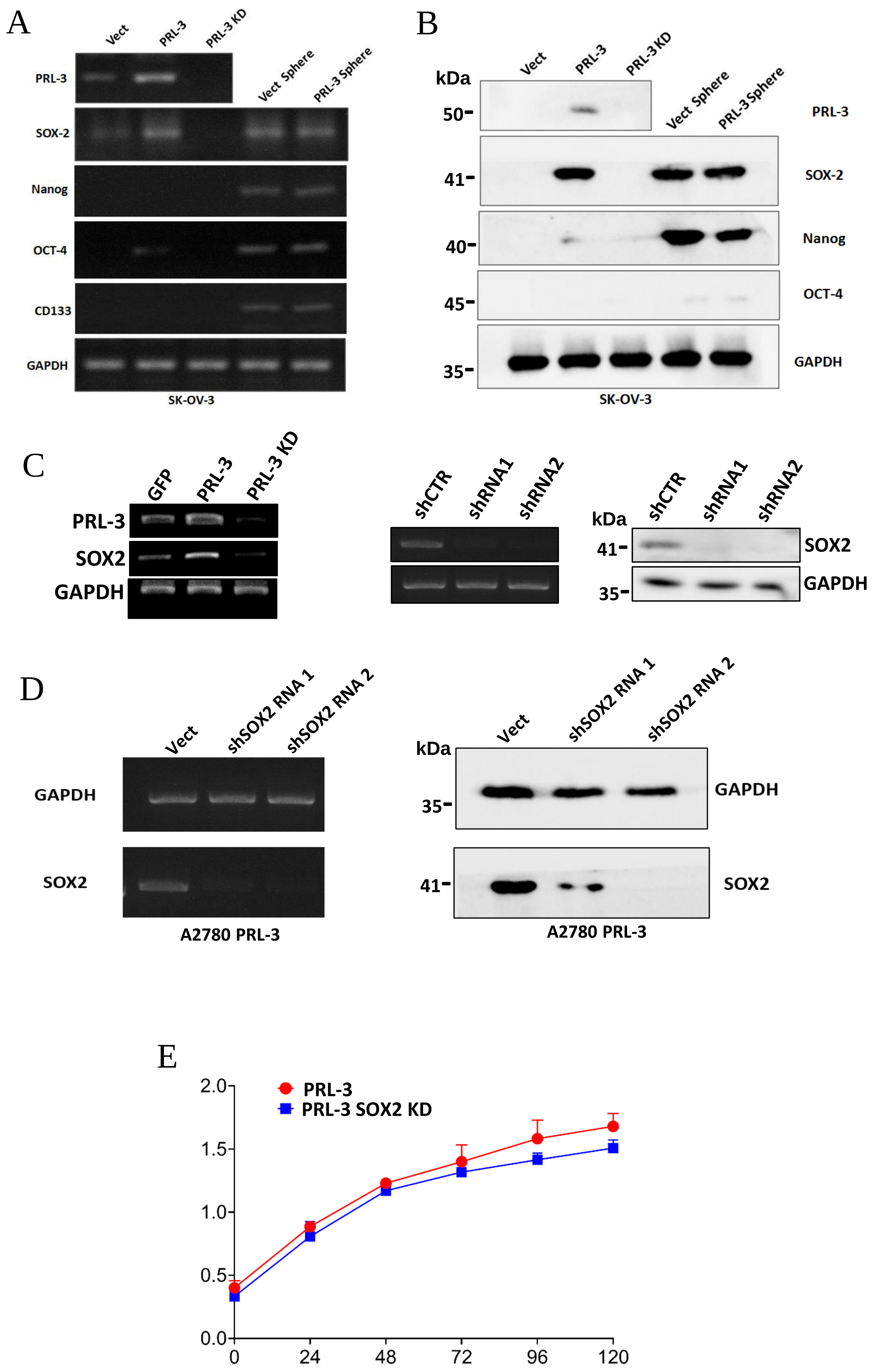
A
Vect
PRL-3
PRL-3 KD
Vect Sphere
PRL-3 Sphere
PRL-3
SOX-2
Nanog
OCT-4
CD133
GAPDH
SK-OV-3
B
kDa
Vect
PRL-3
PRL-3 KD
Vect Sphere
PRL-3 Sphere
50
41
40
45
35
PRL-3
SOX-2
Nanog
OCT-4
GAPDH
SK-OV-3
C
GFP
PRL-3
PRL-3 KD
PRL-3
SOX2
GAPDH
shCTR
shRNA1
shRNA2
kDa
shCTR
shRNA1
shRNA2
41
35
SOX2
GAPDH
D
Vect
shSOX2 RNA 1
shSOX2 RNA 2
GAPDH
SOX2
A2780 PRL-3
kDa
Vect
shSOX2 RNA 1
shSOX2 RNA 2
35
41
GAPDH
SOX2
A2780 PRL-3
E
PRL-3
PRL-3 SOX2 KD
2.0
1.5
1.0
0.5
0.0
0
24
48
72
96
120

Figure S2. PRL-3 upregulates SOX2 expression, Related to Figure 2

A. RT-PCR analyses of the indicated stem cell markers. Total RNA was isolated from SK-OV-3 GFP, SK-OV-3 GFP-PRL-3, SK-OV-3 GFP sphere, and SK-OV-3 PRL-3 sphere. Glyceraldehyde-3-phosphate dehydrogenase (GAPDH) was used as a loading control.
B. Immunoblots of the indicated stem cell markers in A with their specific antibodies.
C. RT-PCR of Sox2 in A2780 GFP and PRL-3 cells transfected with specially designed shRNAs that stably knockdown the expression of PRL-3.
D. Immunoblots and RT-PCR of Sox2 in A2780 cells transfected with specially designed shRNAs that stably knockdown the expression of Sox2.
E. Growth curve of A2780 PRL-3 and A2780 PRL-3 SOX2 KD cells.

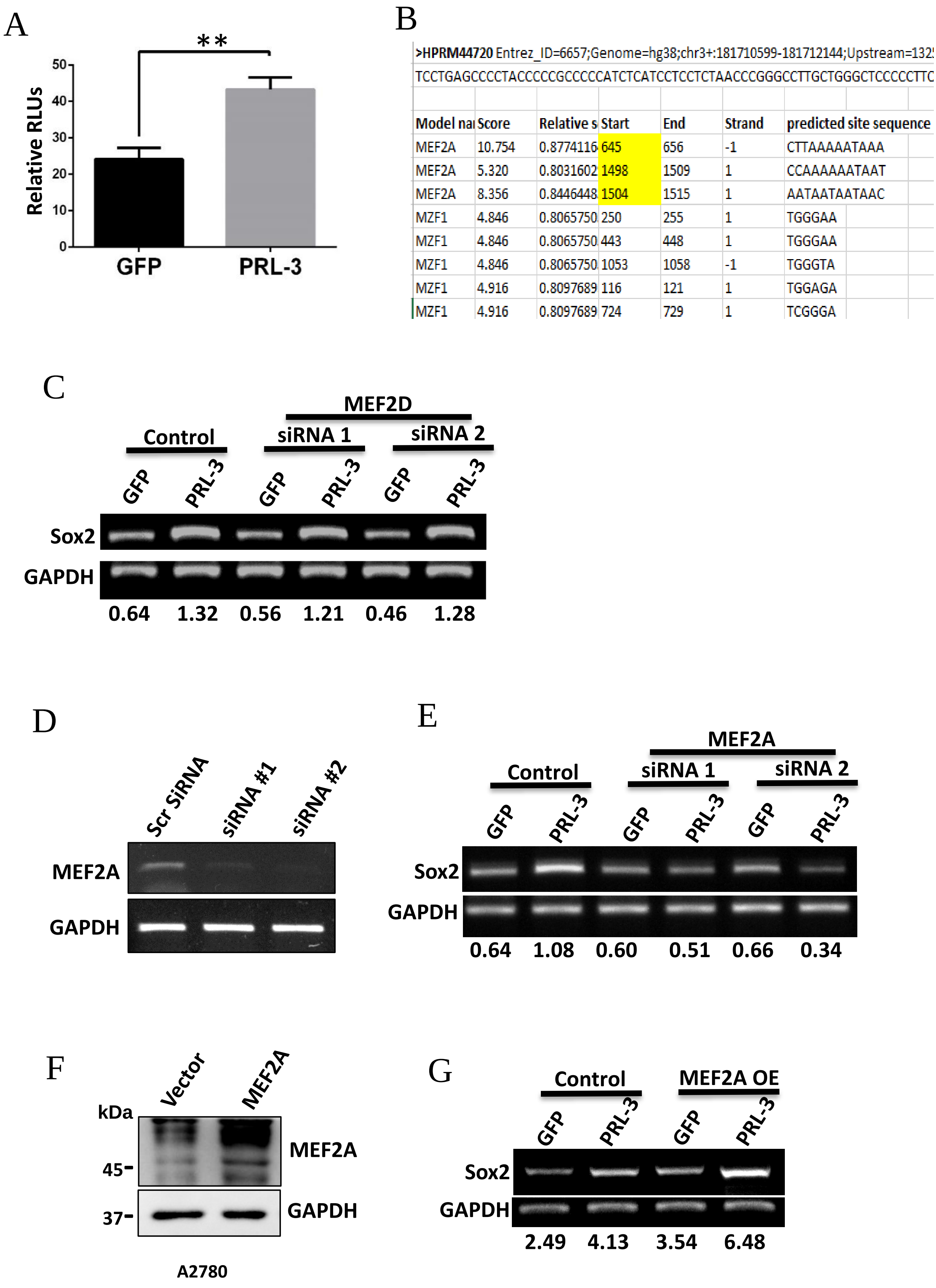
A
**
Relative RLUs
50
40
30
20
10
0
GFP
PRL-3
B
>HPRM44720 Entrez_ID=6657;Genome=hg38;chr3+:181710599-181712144;Upstream=132
TCCTGAGCCCCTACCCCCGCCCCCATCTCATCCTCCTCTAACCCGGGCCTTGCTGGGCTCCCCCTTC
Model na Score Relative s Start End Strand predicted site sequence
MEF2A 10.754 0.8774116 645 656 -1 CTTAAAAATAAA
MEF2A 5.320 0.8031602 1498 1509 1 CCAAAAAATAAT
MEF2A 8.356 0.8446448 1504 1515 1 AATAATAATAAC
MZF1 4.846 0.8065750 250 255 1 TGGGAA
MZF1 4.846 0.8065750 443 448 1 TGGGAA
MZF1 4.846 0.8065750 1053 1058 -1 TGGGTA
MZF1 4.916 0.8097689 116 121 1 TGGAGA
MZF1 4.916 0.8097689 724 729 1 TCGGGA
C
MEF2D
Control
siRNA 1
siRNA 2
GFP
PRL-3
GFP
PRL-3
GFP
PRL-3
Sox2
GAPDH
0.64 1.32 0.56 1.21 0.46 1.28
D
Scr SiRNA
siRNA #1
siRNA #2
MEF2A
GAPDH
E
MEF2A
Control
siRNA 1
siRNA 2
GFP
PRL-3
GFP
PRL-3
GFP
PRL-3
Sox2
GAPDH
0.64 1.08 0.60 0.51 0.66 0.34
F
Vector
MEF2A
kDa
45
37
MEF2A
GAPDH
A2780
G
Control
MEF2A OE
GFP
PRL-3
GFP
PRL-3
Sox2
GAPDH
2.49 4.13 3.54 6.48

Figure S3. MEF2A trancriptionally upregaulates SOX2, Related to Figure 3

A. Luciferase reporter analysis of SOX2 transcription in both HEK 293T cells affected by PRL-3 expression as described in Transparent Methods section. Data are represented as mean ±SEM, **p<0.01, unpaired *t*-test.
B. Possible transcription factors' binding sites on Sox2 promoter region and their scores were predicted. Website: http://biomed.org.ua/COTRASIF/
C. RT-PCR detection of SOX2 in A2780 GFP and PRL-3 cells transfected with 2 siRNAs targeting MEF2D.
D. RT-PCR detection of MEF2A in A2780 cells transfected with siRNAs targeting MEF2A expression.
E. RT-PCR detection of SOX2 in A2780 GFP and PRL-3 cells transfected with siRNAs targeting MEF2A expressions.
F. Immunoblots of MEF2A in A2780 cells transfected with pCGN-MEF2A plasmid.
G. RT-PCR of Sox2 in A2780 GFP and PRL-3 cells transfected with pCGN-MEF2A plasmid.

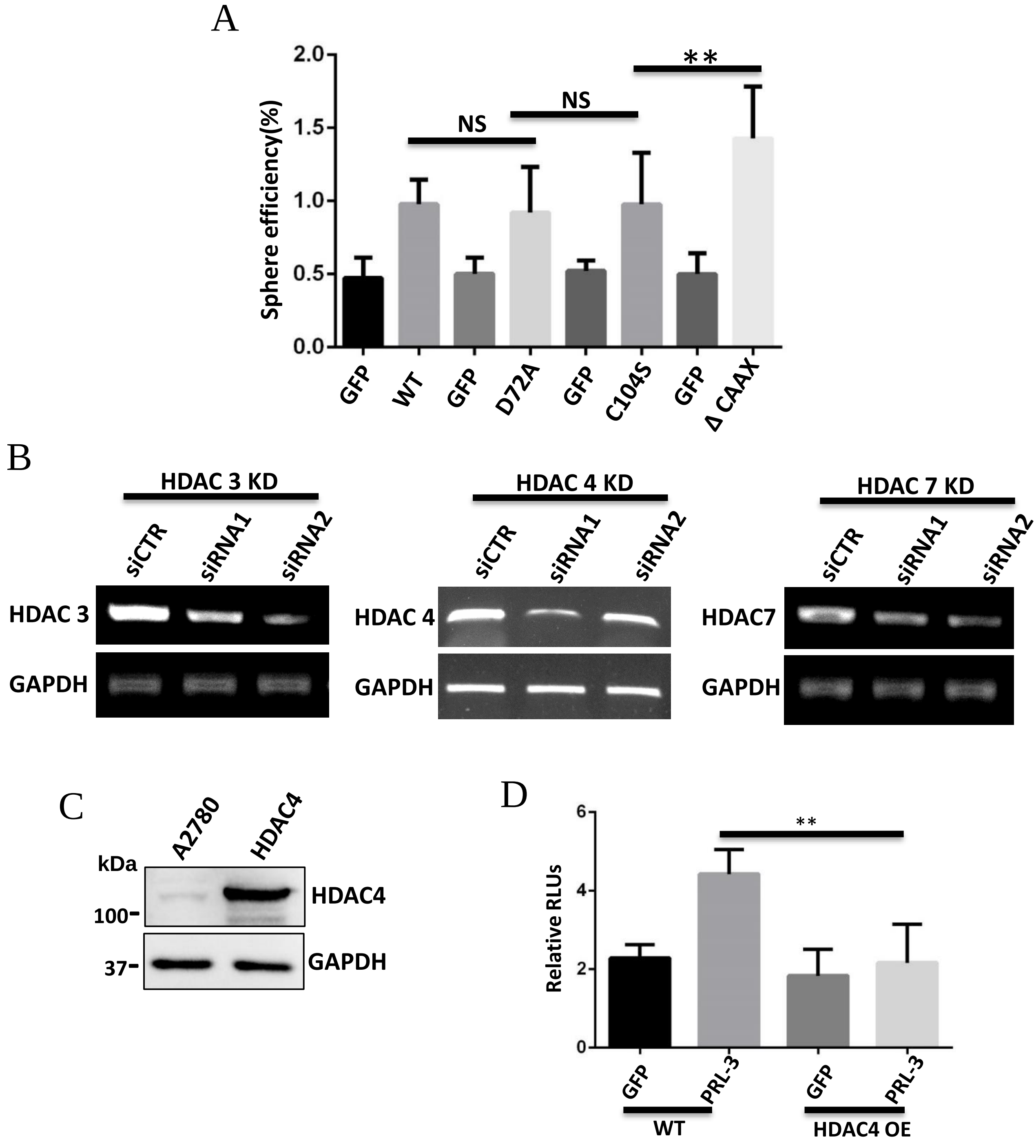

Figure S4 . HDAC4 is involved in PRL-3-mediated tumor sphere formation, Related to Figure 4

A. Sphere efficiency analysis of SOX2 transcription in A2780 cells transfected with wild type (WT) or the indicated PRL-3 mutants. Data are represented as mean ±SEM, **p<0.01, unpaired *t*-test.

B. RT-PCR detection of HDAC3, HDAC4 and HDAC7 in A2780 cells transfected with siRNAs targeting HDAC3, HDAC4, HDAC7 or scrambled control siRNAs. Total RNA was extracted and analyzed with RT-PCR to examine the expression of HDAC3, HDAC4 and HDAC7.

C. Immunoblots of HDAC4 in A2780 cells transfected with pcDNA-HDAC4-FLAG plasmid.

D. Luciferase reporter analysis of SOX2 transcription in A2780 GFP and PRL-3 cells transfected with pcDNA-HDAC4-FLAG vector. Data are represented as mean ±SEM, **p<0.01, unpaired *t*-test.

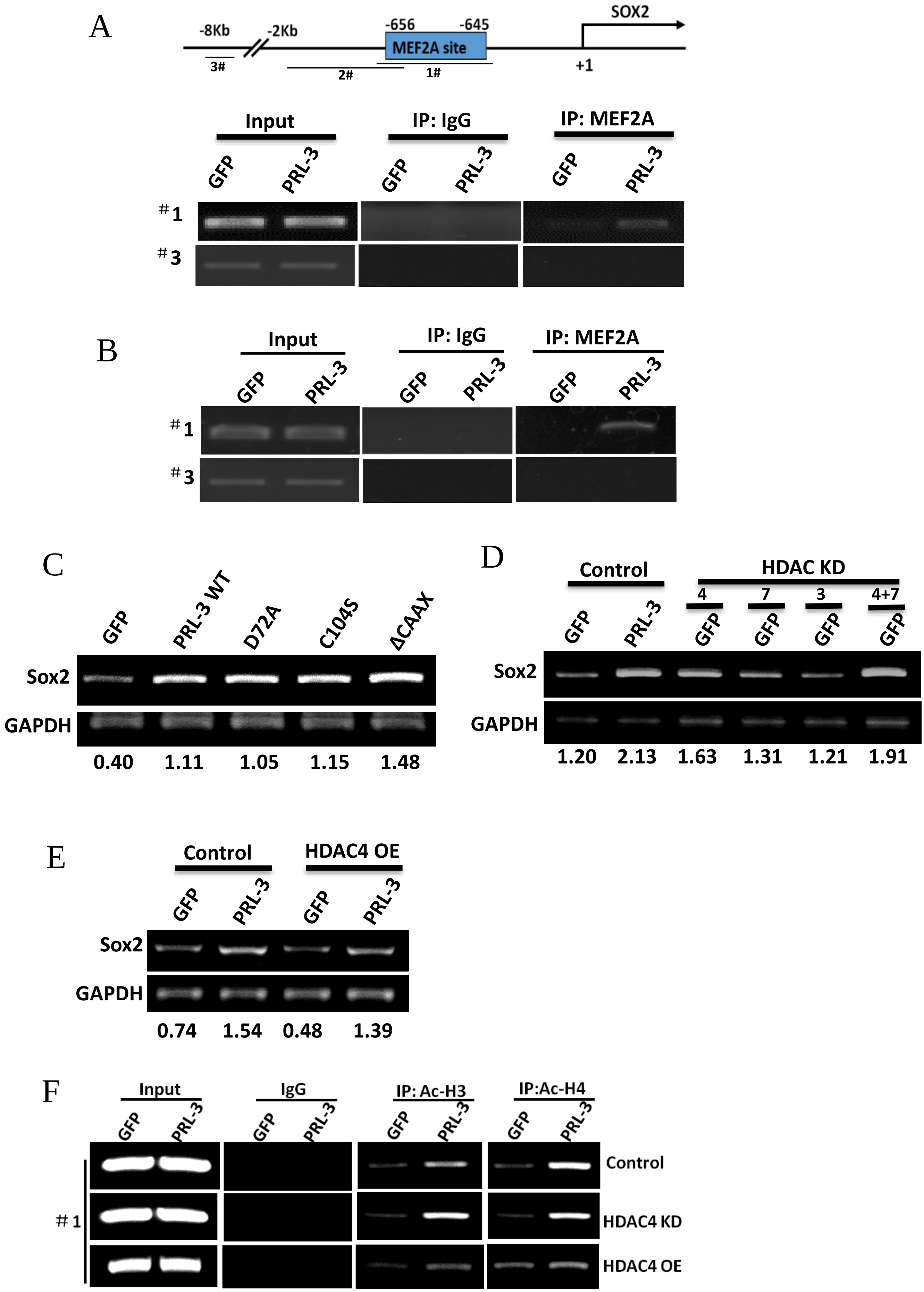

A
-8Kb
-2Kb
-656
-645
MEF2A site
SOX2
+1
3#
2#
1#
Input
IP: IgG
IP: MEF2A
GFP
PRL-3
#1
#3
B
Input
IP: IgG
IP: MEF2A
GFP
PRL-3
#1
#3
C
GFP
PRL-3 WT
D72A
C104S
ΔCAAX
Sox2
GAPDH
0.40 1.11 1.05 1.15 1.48
D
Control
HDAC KD
4
7
3
4+7
GFP
PRL-3
Sox2
GAPDH
1.20 2.13 1.63 1.31 1.21 1.91
E
Control
HDAC4 OE
GFP
PRL-3
Sox2
GAPDH
0.74 1.54 0.48 1.39
F
Input
IgG
IP: Ac-H3
IP:Ac-H4
GFP
PRL-3
#1
Control
HDAC4 KD
HDAC4 OE

Figure S5. HDAC4 modulates MEF2A accessibility to SOX2 promoter, Related to Figure 5

A. Chromatin immunoprecipitation of MEF2A-bound SOX2 promoter fragments in both A2780 cells affected by PRL-3. The diagram of Sox2 promoter regions and the fragments for detection are indicated. The MEF2A binding site (fragment #1), negative far upstream fragment (#3) were examined by semi-quantitative RT–PCR.
B. Chromatin immunoprecipitation of MEF2A-bound SOX2 promoter fragments in both 293T and 293T PRL-3 cells as in A.
C. RT-PCR detection of SOX2 mRNA levels in A2780 GFP and PRL-3 cells transfected with wild type or the indicated PRL-3mutants.
D. RT-PCR detection of SOX2 mRNA in A2780 GFP transfected with siRNAs targeting HDAC3, HDAC4, HDAC7 expressions and PRL-3 cells.
E. RT-PCR detection of SOX2 in A2780 GFP and PRL-3 cells upon HDAC4 overexpression.
F. Chromatin immunoprecipitation of the acetylation state of SOX2 promoter region by the indicated acetylated histone 3 (AcH3), or histone 4 (AcH4) in both A2780 GFP and PRL-3 cells, with HDAC4 knockdown (KD) and overexpression (OE), respectively.

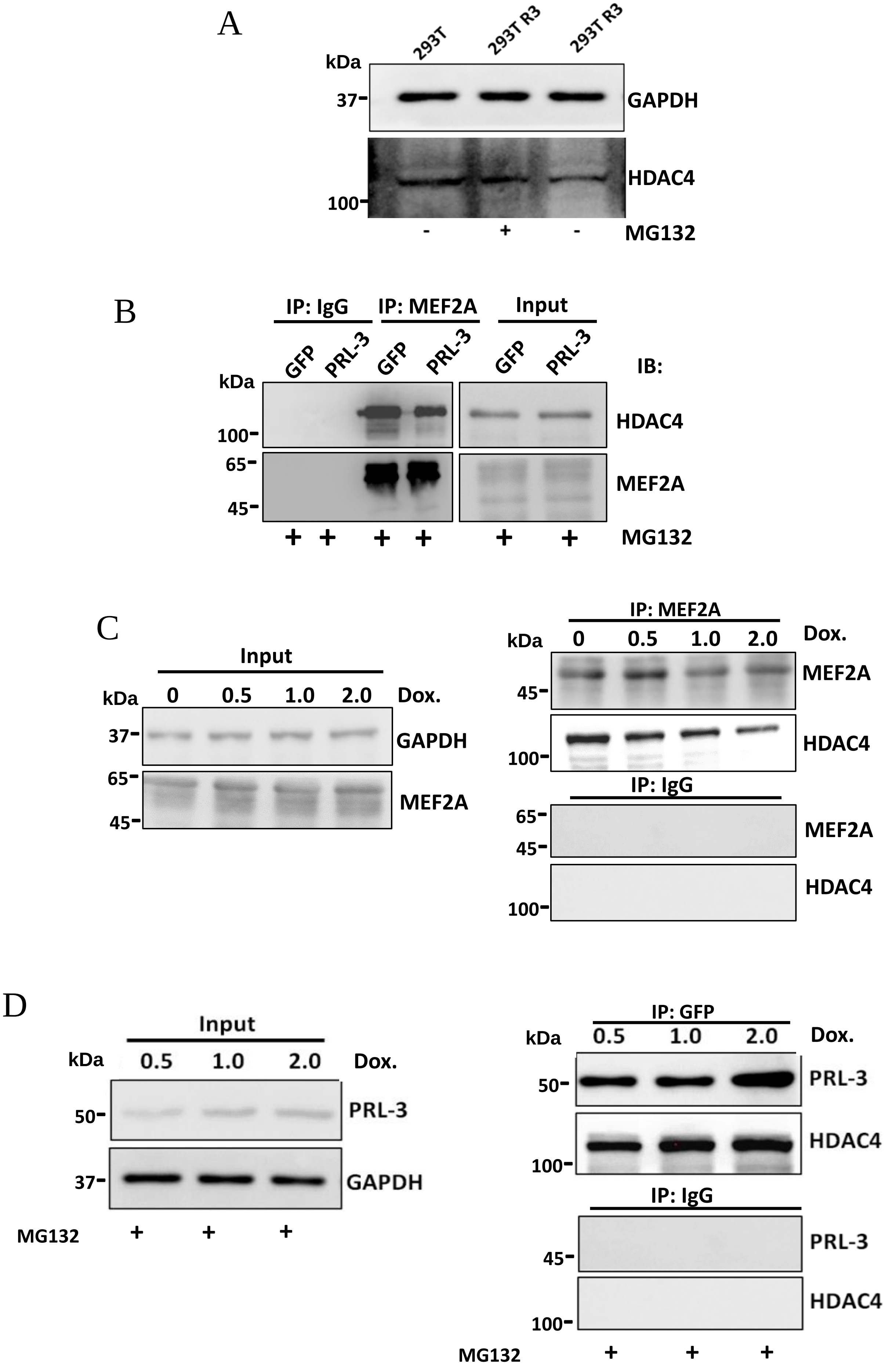

A
293T
293T R3
293T R3
kDa
37
GAPDH
HDAC4
100
-
+
-
MG132
B
IP: IgG
IP: MEF2A
Input
GFP
PRL-3
GFP
PRL-3
GFP
PRL-3
IB:
kDa
HDAC4
100
65
MEF2A
45
+
+
+
+
+
+
MG132
C
Input
kDa
0
0.5
1.0
2.0
Dox.
37
GAPDH
65
MEF2A
45
IP: MEF2A
kDa
0
0.5
1.0
2.0
Dox.
MEF2A
45
HDAC4
100
IP: IgG
65
MEF2A
45
HDAC4
100
D
Input
kDa
0.5
1.0
2.0
Dox.
PRL-3
50
37
GAPDH
MG132
+
+
+
IP: GFP
kDa
0.5
1.0
2.0
Dox.
50
PRL-3
HDAC4
100
IP: IgG
PRL-3
45
HDAC4
100
MG132
+
+
+

Figure S6. PRL-3 competitively binds to HDAC4 for its degradation, Related to Figure 6

A. Immunoblots of HDAC4 in 293T cells with PRL-3 expression (R3) with or without MG132 treatment.

B. Immunoprecipitation of HDAC4 in both A2780 GFP and A2780 GFP-PRL-3 cells with MG 132 pre-treatment. Binding of HDAC4 to MEF2A was examined by immunoblotting (IB).

C. Immunoprecipition of HDAC4 by MEF2A along with the induction of PRL-3 in CHO cells. Total cell lysates (Input) and precipitated proteins were detected by immunoblots (IB). IgG was used as a negative control for immunoprecipitation.

D. Immunoprecipitations of HDAC4 with PRL-3 in PRL-3-inducible expression 293T cells. Binding amounts of HDAC4 to PRL-3 was examined by immunoblotting (IB).

Supplemental Table S1. Primers used in this study, Related to Figure 2 and Figure 4

Target	Forward	Reverse
PRL-3	5’-TACAAACACATGCGCTTCCTC-3’	5’- CTGTTTGGGCCGGTATTTCT-3’
SOX2	5’-CATGGACAGTTACGCGCACA-3’	5’-CCCTCCCATTTCCCTCGTTT-3’
OCT4	5’ GACAACAATGAGAACCTTCAGGAGA- -3’	5’- CTGGCGCCGGTTACAGAACCA-3’
Nanog	5’-GTCTTCTGCTGAGATGCCTCACA-3’	5’- CTTCTGCGTCACACCATTGCTAT-3’
CD133	5’-AAGCATTGGCATCTTCTATGG-3’	5’- AAGCACAGAGGGTCATTGAGA-3’
MEF2A	5’-TGCGGAATCATAAAATCGCACC-3’	5’- GGACTGCTTCCAGCTCCATT-3’
MEF2B	5’-GACCGTGTGCTGCTGAAGTA-3’	5’- AGCGTCTCGAGGATGTCAGT-3’
MEF2C	5’-TCCACCTCCCAGCTTTGAGAT-3’	5’- TGCCAGGTGGGATAAGAACG-3’
MEF2D	5’-GGGGGTGACCTGAACAGTGC-3’	5’- GTGATGACTCGCAGGTCGGG -3’
HDAC4	5’-GGTGGTGTTGGGGTGGACAG-3’	5’- GCTCTCCTCCGCATGGTGTC-3’
HDAC3	5’-CCCTGCGGGATGGCATTGAT-3’	5’- GGCCTCTTCTACCAGCAGCG-3’
HDAC5	5’-TGTGACAGTGGAGGTGAAGC-3’	5’- GTGGGAGGGAATGGTTGAGG-3’
HDAC7	5’-TGCCGACAGTGTCCTTGCTG-3’	5’- ACTGGGCAAAGTGGAAGGGC-3’
HDAC9	5’-CTGGGCAGTATGGAGGCAGC-3’	5’- GGCAAAGGTGCAGACTGGGT-3’
1#	5’-GGTTCTCAGCTCTAGAGTCTGCC -3’	5’- CTTCCTTGCTTCCACGTAACTTGC -3’
2#	5’-GTTTGAGCCCCAGGCTTAAGCC -3’	5’- CTTCCCTCCTCCTCTGGCCG-3’
3#	5’-CGTGCAATAGCAGAGTCCTGG -3’	5’- CAACAACCTGCTACCCCACAGGC -3’
GAPDH	5’-CGGAGTCAACGGATTTGGTCGTAT-3’	5’-AGCCTTCTCCATGGTGGTGAAGAC-3’

Transparent Methods

Cell culture and sphere formation assay

Human A2780 ovarian cancer cells, Human SK-OV-3 ovarian cancer cells, Chinese Hamster Ovary (CHO) cells were purchased from American Type Culture Collection (ATCC, Rockville, MD) and maintained in RPMI-1640 medium supplemented with 10% heat-inactivated fetal bovine serum and 1% antibiotics (Sigma) at 37 ^{o}C with 5% CO_2. For sphere induction formation assay, we added the recombinant EGF (10 ng/ml) and bFGF (5 ng/ml) to DMEM/F12 medium with B27 as supplement, namely as sphere culture medium. When induction of tumor cell sphere, we used trypsin to detach the proper cultured adhesion cells to prepare the single cell suspension, and seeded 5000 cells from the suspensions into each well of 6-well plates containing 2 ml sphere culture medium. Meanwhile, 0.4% (weight/volume) of poly 2-hydroxyethyl methacrylate (pHEMA) dissolved in 95% ethanol was added into the each well for blockade of cell attachment to plates. After 5 days, sphere efficiency was calculated as (sphere numbers*100/5000) %.

Plasmids construction and cell transfection

The pEGFP-PRL-3, PRL-3 mutants and PRL-3 knockdown shRNA expression plasmids were constructed as previously described (Qu et al. 2014). The dose-dependent PRL-3 induction expression system with doxycycline was constructed as before (Zeng et al. 1998). PCGN-MEF2A (Cat No. #32958) and pcDNA-HDAC4-FLAG (Cat. No. #30485) plasmids were acquired from Addgene. For knockdown of SOX2, the specific shRNAs were synthesized and constructed into the same vector as those of PRL-3 knockdown shRNAs, just replacing PRL-3-targeted sequences with "CTGCCGAGAATCCATGTATAT" or "AGGAGCACCCGGATTATAAAT". For the luciferase analysis, SOX2 reporter construct was purchased from GeneCopoeia Inc., in which 1546 bp of SOX2 promoter region fused to luciferase-encoding gene. The indicated serial of the deleted mutants of the SOX2 promoter were generated by PCR method, following the QuikChange™ Site-Directed Mutagenesis Kit protocol (Agilent, CA, USA) with specifically designed primers (Supplementary Table S1). Assayed cells were tranfected with Lipofectamine® 2000 Reagent (Invitrogen) following the supplier's manual instruction, except for 293T cells with calcium chloride.

Luciferase reporter assay

Cells in 6-well plate were transiently transfected with 3 μg of the full-length or the indicated deletion mutants of SOX2 promoter-luciferase constructs. After 24 hours of transfection, 1 ml medium from each well was collected and measured with Secrete-Pair™ Dual Luminescence Assay Kit (GeneCopoeia), according to the supplied manual.

siRNA oligos

The small interference RNAs (siRNAs) of MEF2A and MEF2D, HDAC3, HDAC4, and HDAC7 were synthesized by synbio-tech.

Semi-and quantitative RT-PCR

Total RNA was extracted from cells using Qiagen RNeasy kit (QIAGEN), following the manufacturer's instructions. cDNAs were synthesized with SuperScript™ II Reverse Transcriptase (Invitrogen) with Oligo dT at 42 ^{o}C for 1 hour, with the extracted total RNA as template. GAPDH amount was used as an internal loading control. Image J software was used to quantitatively analyze the relative expression levels of target genes. The primers used in experiments are listed in supplementary data (Table S1).

Cellular fraction of cytosol and nuclear proteins
Cells were washed with 1 ml of ice-cold PBS, harvested and suspended in the cellular fraction reagent A (Beyotime). After Votex briefly, cells were put on ice for 15 minutes. Cellular fraction reagent B was added to the mixture and centrifuged at 12,000 g for 5 minutes at 4°C. The sediment contains the nuclei, while the supernatant contains cytosol protein. Further, the nuclei pellet was mixed with RIPA protein extraction reagent (Genestar) for 30 min (votex every 20 seconds) and centrifuged at 10,000 g for 10 min at 4 °C to collect the supernatant as nuclear protein.
Antibodies
Antibodies used were as follows: anti-GAPDH Rabbit mAb (#2118, Cell Signaling Technology), anti-Sox2 rabbit polyclonal antibody (11064-1-AP, Proteintech), anti-Nanog (1E6C4)(sc-293121, Santa Cruz Biotechnology), anti-OCT4 antibody (#2750, Cell Signaling Technology), anti-IgG (3E8)(sc-69786, Santa Cruz Biotechnology), anti-MEF2A mouse monoclonal antibody (sc-17785, Santa Cruz Biotechnology), anti-HDAC4 rabbit polyclonal antibody (17449-1-AP, Proteintech), anti-acetylated-Lysine antibody (#9441, Cell Signaling Technology), anti-GFP rabbit mAb (#2956, Cell Signaling Technology), anti-Ubiquitin Antibody (#3933, Cell Signaling Technology), anti-PRL-3 mouse monoclonal antibody (sc-130355, Santa Cruz Biotechnology).
Immunofluorescence staining and Immunohistochemistry
For Immunofluorescence, cells less than 50% of confluency were seeded and grown on coverslips overnight. Spheres were precipitated and washed according to Sasaki's method (Sasaki et al. 2010). Cells or spheres were fixed with 4% paraformaldehyde and permeabilized with Triton-100. After blocking in 5% goat serum, samples were incubated with the appropriately diluted primary and secondary antibodies. Mouse IgG antibody (same IgG1 Kappa light chain as Nanog mouse antibody from Santa Cruz) was used as an isotype negative control. Eventually, cells were observed and photographed by fluorescence microscopy (Nikon C2). For immunohistochemistry, the paraffin-embedded tissues were sectioned into slices in 5-7 μm thickness with a microtome (Leica), and mounted onto the adhesive microscope slides in warm water (40°C). Sections were allowed to dry overnight at room temperature. After deparaffinization and rehydration, sections were subjected to the specific primary antibody incubation overnight at 4°C. Sections were then incubated and stained using PV-6000 goat anti-mouse or rabbit IHC kit (Zsbio). Sections were examined and the photos were captured with an inverted fluorescent microscope (Nikon). The staining intensity was scored with the following rules: 0, <10%, 1, 10-25%, 2, 25-50%, 3, 50-75%, 4, >75%. Clinical samples were divided into two groups: PRL-3 low (with score 0, 1 and 2) and PRL-3 high (with score 3 and 4).
Co-Immunoprecipitation and Western Blotting
Cells with 70-85% of confluency were washed with ice-cold PBS and lysed in cold lysis buffer (10 mM Tris-HCl, pH 7.4, 150 mM NaCl, 1% Triton X-100, 0.5% NP-40, 1mM EDTA, 0.2 mM Sodium orthovanadate, 0.2 mM PMSF and protease inhibitor cocktail) on ice for 30 min. Total lysates were collected with rubber scrapers into tubes on ice, and then clarified at 14,000 rpm for 15 min at 4 ºC. Total protein concentration was determined by BCA protein assay (Bio-Rad). For co-immunoprecipitation (Co-IP), Pierce® Crosslink Immunoprecipitation Kit was used accordingly. Briefly, the clarified cell lysates were incubated with protein G-agarose beads cross-linked with respective antibody (2 μg) or mouse IgG (30000-0-AP, Proteintech), overnight at 4°C on a rotator. After incubation, the bound proteins were washed extensively with lysis buffer, prior to boiling in 2 x SDS loading buffer for loading analysis. For western blotting, total lysates or IP eluates were run on a 10% SDS-PAGE gel, and transferred into a PVDF membrane (Amersham). The blots were blocked in 5% milk in PBS with 0.01% Tween 20 for 2 hours.

After incubation with primary antibodies for overnight at 4 ^{o}C, blots were washed and incubated with HRP-labeled anti-rabbit (Cell Signaling Technology) or anti-mouse antibody (Cell Signaling Technology) for 2 h and developed using an enhanced chemiluminescence kit (Pierce). Images were acquired by ChemiDoc™ Touch Imaging System (Bio-Rad).

Chromatin immunoprecipitation

Chromatin immunoprecipitation was performed according to the method described by Nelson (Nelson et al. 2006) with slight modifications. In brief, cells were fixed with formaldehyde and collected. After sonication, cell lysates were incubated with protein A+G agarose beads and specific antibodies respectively. After incubation, beads were carefully collected and washed for several times. The bounded DNA was isolated by boiling and precipitated.

In vivo xenograft tumor formation assay

Monolayer (adhesion cells) or tumor sphere cells (dispersed) were trypsinzied and suspended in PBS/Matrigel mixture (2:1 in v/v), followed by implantation of 0.2 ml of this mixture subcutaneously (s.c.) into flanks of 8-week-old female NOD/SCID mice (The Jackson Lab). The inoculated mice were well feed to record tumor formation and progression, until the tumor sizes were apparent or to a limit of 1.5 cm^3 in volume. At end of experiments, the mice were humanely sacrificed. All animal studies were approved by the Institutional Animal Care and Use Committee (IACUC) and were carried out under the policies of Sun Yat-sen University, China.

Aldefluor Assay for FACS

The ALDEFLUOR kit (StemCell Technologies, Catalog #01700) was used to analyze the population with a high ALDH enzymatic activity based on Ginestier's research (Ginestier et al. 2007). 10^6 PRL-3^+ or PRL-3^- cells were suspended in 1 ml ALDEFLUOR assay buffer containing 5 µl ALDH substrate (BAAA) and incubated for 40 min at 37 degree. As negative control, the same number of cells were treated with 5 µl ALDH substrate (BAAA) and incubated for 40 min at 37 degree. As negative control, the same number of cells were treated with 5 µl diethylaminobenzaldehyde (DEAB), a specific ALDH inhibitor. The sorting was established using gate on all nucleated cells to exclude RBCs and debris (R1), then gate the rightmost edge of the stained DEAB control population with the second log decade on the FL1 axis (R2). Then analyze the corresponding sample tubes with the above gate R2.

Cell Proliferation Assay

Cell Counting Kit-8 (Dojindo) was used to analyze the proliferation of A2780 PRL-3 or A2780 PRL-3 SOX2 KD cells. Dispense 100 µl of cell suspension (5000 cells/well) in a 96-well plate. Incubate the plate for an appropriate length of time (24, 48, 72 ,96 or 120 hours) in the incubator (at 37°C, 5% CO_2). Add 10 µl of CCK-8 solution to each well of the plate and Measure the absorbance at 450 nm using a microplate reader (TECAN).

TCGA data mining and statistics

The GraphPad Prism 6.0 were adopted to perform the statistical analysis and the statistical data are presented as mean ± SEM. Comparisons between groups were analyzed using Student's t-test. The TCGA data acquired from GEPIA (http://gepia.cancer-pku.cn/detail.php?clicktag=correlation#iframe). The correlation between PRL-3, SOX2 and HDAC4 were assessed using Spearman correlation analysis. Differences were considered to be statistically significant with *$p<0.05$, **$p<0.01$, and ***$p<0.001$, respectively. Sox2 and HDAC4 expression levels in the PRL-3-high and -low groups were respectively compared with unpaired *t*- test.

Supplemental References

Qu, S., Liu, B., Guo, X., Shi, H., Zhou, M., Li, L., Yang, S., Tong, X. and Wang, H. (2014) 'Independent oncogenic and therapeutic significance of phosphatase PRL-3 in FLT3-ITD-negative acute myeloid leukemia', *Cancer*.

Zeng, Q., Tan, Y. H. and Hong, W. (1998) 'A Single Plasmid Vector (pSTAR) Mediating Efficient Tetracycline-Induced Gene Expression', *Analytical Biochemistry,* 259(2), 187-194.

Sasaki, R., Aoki, S., Yamato, M., Uchiyama, H., Wada, K., Ogiuchi, H., Okano, T. and Ando, T. (2010) 'A protocol for immunofluorescence staining of floating neurospheres', *Neurosci Lett,* 479(2), 126-127.

Nelson, J. D., Denisenko, O. and Bomsztyk, K. (2006) 'Protocol for the fast chromatin immunoprecipitation (ChIP) method', *Nat Protoc,* 1(1), 179-85.

Ginestier, C., Hur, M. H., Charafe-Jauffret, E., Monville, F., Dutcher, J., Brown, M., Jacquemier, J., Viens, P., Kleer, C. G., Liu, S., Schott, A., Hayes, D., Birnbaum, D., Wicha, M. S. and Dontu, G. (2007) 'ALDH1 Is a Marker of Normal and Malignant Human Mammary Stem Cells and a Predictor of Poor Clinical Outcome', *Cell Stem Cell,* 1(5), 555-567.
